# Comparative metagenomics reveals expanded insights into intra- and interspecific variation among wild bee microbiomes

**DOI:** 10.1038/s42003-022-03535-1

**Published:** 2022-06-17

**Authors:** Wyatt A. Shell, Sandra M. Rehan

**Affiliations:** Department of Biology, York University, Toronto, ON Canada

**Keywords:** Molecular ecology, Microbial ecology

## Abstract

The holobiont approach proposes that species are most fully understood within the context of their associated microbiomes, and that both host and microbial community are locked in a mutual circuit of co-evolutionary selection. Bees are an ideal group for this approach, as they comprise a critical group of pollinators that contribute to both ecological and agricultural health worldwide. Metagenomic analyses offer comprehensive insights into an organism’s microbiome, diet, and viral load, but remain largely unapplied to wild bees. Here, we present metagenomic data from three species of carpenter bees sampled from around the globe, representative of the first ever carpenter bee core microbiome. Machine learning, co-occurrence, and network analyses reveal that wild bee metagenomes are unique to host species. Further, we find that microbiomes are likely strongly affected by features of their local environment, and feature evidence of plant pathogens previously known only in honey bees. Performing the most comprehensive comparative analysis of bee microbiomes to date we discover that microbiome diversity is inversely proportional to host species social complexity. Our study helps to establish some of the first wild bee hologenomic data while offering powerful empirical insights into the biology and health of vital pollinators.

## Abstract (web summary)

Global wild bee metagenomes provide insights into microbiome, sociality and pollinator health.

## Introduction

Bees are a highly diverse and critical group of pollinators, represented by over 20,000 species worldwide^1,2^, which contribute an estimated value of at least $200 billion in agricultural services per year^3^. The ongoing and alarmingly pervasive decline in bee populations around the globe thus represents a major ecological and economic issue. Bee health and fitness are strongly affected by the bacteria, fungi, and viruses they are exposed to in their environments;^4,5^ and there is evidence that both beneficial and harmful microbiota may be regularly transmitted within and between species^4–10^. Recent studies exploring hologenomic data consider the genomes of a host species and its microbiome in concert. These works indicate that host species may be continuously coevolving with their microbial communities^11^. As such, hologenomic data can be used to achieve insights into a host species and its environmental ecology simultaneously^12^. Accordingly, alongside studies of bee behavior, genetics, ecology, and evolution, research into bee microbiomes has begun to reveal an intimate loop of influence between microbes, bees, and their shared ecosystems^12–16^.

To date, microbiome studies have primarily focused on the gut microbiomes of economically salient bee groups, such as honey bees (genus *Apis*) or bumble bees (genus *Bombus*), applying targeted amplicon sequencing methods to identify bacterial communities and make inferences regarding their influences on their hosts^10,17^. We now appreciate that the microbiomes of highly social honey bees and other members of the subfamily Apinae (including bumble bees, stingless bees, and orchid bees), collectively termed corbiculate bees, are composed of relatively small suites of bacteria, which nonetheless provide major benefits through facilitating nutrient uptake and immune functions for their hosts^18–20^. Similar research among wild bees—which provide the majority of pollination services^21^—promises to offer similarly invaluable insights despite remaining in its early stages^16^. For example, we are gaining an appreciation for the importance of pollen-borne microbes for wild bee development and fitness^13,22^ and learning that the composition of microbial communities within plant-pollinator networks is likely highly dynamic^23^.

Ongoing advancements in next generation sequencing methods have allowed for the production of metagenome data—offering massively expanded insights into the environmental elements potentially influencing organismal health and biology^12,24–27^. Compared to the more targeted efforts of 16S sequencing, metagenomic datasets capture a wholistic profile of bacteria, fungi, viruses, plants, and other taxa, associated with host organisms at the time of sampling. To date, these methods have been applied primarily to honey bees^12,25–29^, allowing for fuller characterization of host microbiomes alongside identification of potentially pathogenic elements^26,29^. Other studies have focused on potential roots of honey bee disease. For example, recent efforts by Galbraith et al.^25^ identified a suite of bee viruses, including evidence of 127 novel viral contigs, from both managed and wild bee species around the globe; an important finding considering previous metagenomic research revealed that honey bee viruses may be readily circulated by wild bee populations^28^. These studies also highlight a paucity of metagenomic data from any wild bees beyond the corbiculates, data that promises to help capture a more complete picture of wild bee health and ecology.

One particularly suitable candidate group for this work are the *Ceratina* small carpenter bees, a well-studied wild bee genus which is globally distributed and features considerable social diversity among its members^30,31^. All *Ceratina* form small burrows in the soft pith of woody-stemmed plants (especially of *Rubus* or *Rhus* spp.) in which they usually rear a single brood per year^31^. This form of stem nesting makes the *Ceratina* highly tractable for empirical study, and three species (*C. japonica*, *C. calcarata*, and *C. australensis*) have already emerged as powerful models for research into behavioral ecology^32,33^, sociogenomics^34–37^, and the combined influences of both nutrition and microbial composition on development and behavior^14,38,39^. Generation of metagenomic data for these species in particular would provide novel insights into wild bee ecology while greatly advancing the comparative study of bee health overall.

Here, we present the metagenomes of three globally distributed species of *Ceratina*, *C. australensis* (Australia;^35^), *C. japonica* (Japan;^32^), and *C. calcarata* (North America;^34^), and use these data to address four focal questions: (1) Do metagenomic data characterize host *Ceratina* species? We hypothesize that metagenomes will be largely unique to hosts. *Ceratina* likely do not feature sufficient sociality to standardize their microbiome (as seen among corbiculates;^20^); rather, these three species are likely strongly affected by the microbiota of their local environments^7–9^ and should therefore reflect their highly isolated and distinct environments in their metagenomes. (2) Does local environment drive metagenomic composition among populations of *C. australensis* across its distribution? We hypothesize that variation among *C. australensis* metagenomes is directly tied to environmental distinction among populations, as has been seen in previous amplicon sequencing microbiome studies within the species^40,41^. (3) Does the *C. australensis* metagenome reveal signals of sociality? Previous research has shown that about 13% of *C. australensis* females demonstrate a form of cooperative breeding across their distribution^42^, but that selection on social traits may be highly population-specific and spread across many genetic loci^43^. We thus hypothesize that any metagenomic influence associated with social phenotypes in the species will be tied to local population rather than consistently distributed across populations. (4) How does the core bacterial microbiome of *Ceratina* compare to the microbiomes of other bee species drawn from across a globally distributed spectrum of bee families and social forms? We hypothesize that, despite considerable social polymorphism within the group^37^, our largely solitary *Ceratina* species are less able to mitigate their exposure to microbial species from their environment. As such, we predict the core *Ceratina* microbiome will share more elements with those of other solitary and weakly social bees from distantly related families than they will with their sister subfamily of the highly social corbiculate bees^20^. This study defines the first core carpenter bee microbiome and offers the most comprehensive comparative metagenomic assessment of wild bees to date. As the genomes of the featured taxa have also been recently sequenced^34,35,37^, the metagenomic data presented here also represents the completion of the first wild bee hologenomes. Overall, this study thus provides an exciting and highly encouraging demonstration of the power of metagenomic methods to yield a wealth of insights into wild bee ecology and health.

## Results

### Metagenomic data characterizes host species

A total of 221 families (40% of all 556 families identified) were hosted in each of the *Ceratina* species we measured. This set of 221 families contained prominent core taxa shared across the *Ceratina* genus, including the bacterial genera *Burkholderia*, *Bacillus*, *Paenibacillus* and *Lactobacillus* (Data S3). This set also included notable virus families (e.g., Potyviridae, Secoviridae, and Retroviridae) and mite genera (e.g., *Varroa*).

**Fig. 1:**
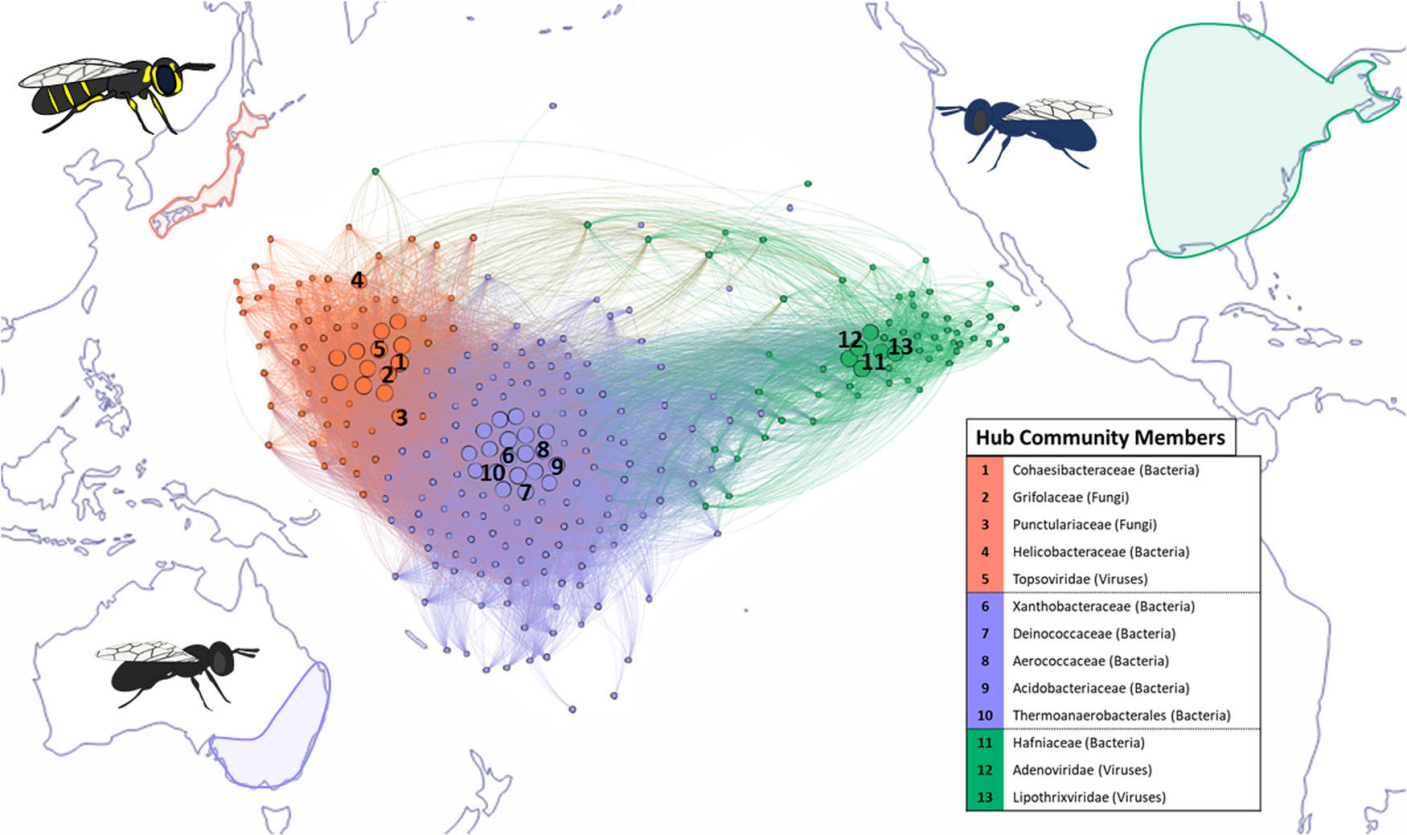
Microorganismal community co-occurrence network among host *Ceratina* species. Results of weighted gene co-expression network analysis (WGCNA) showing the topmost strongly positively and significantly correlated modules for each host species (full outputs in Data S9–S11) overlaid on a world map outlining endemic distributions of each host. A selection of bacterial, fungal, or viral hub families, which co-occur extensively with other members of their micro-communities, are highlighted (see legend). The featured *Ceratina australensis* module (cor = 0.99, *p* = 7.2e−139) was found to be predominantly composed of bacterial families (53%), compared to the more diverse core communities of *C. calcarata* (cor = 0.97, *p* = 8.4e−27; bacteria = 19%) and *C. japonica* (cor = 1.0, *p* < 1.0e−200; bacteria = 13%).

**Fig. 2:**
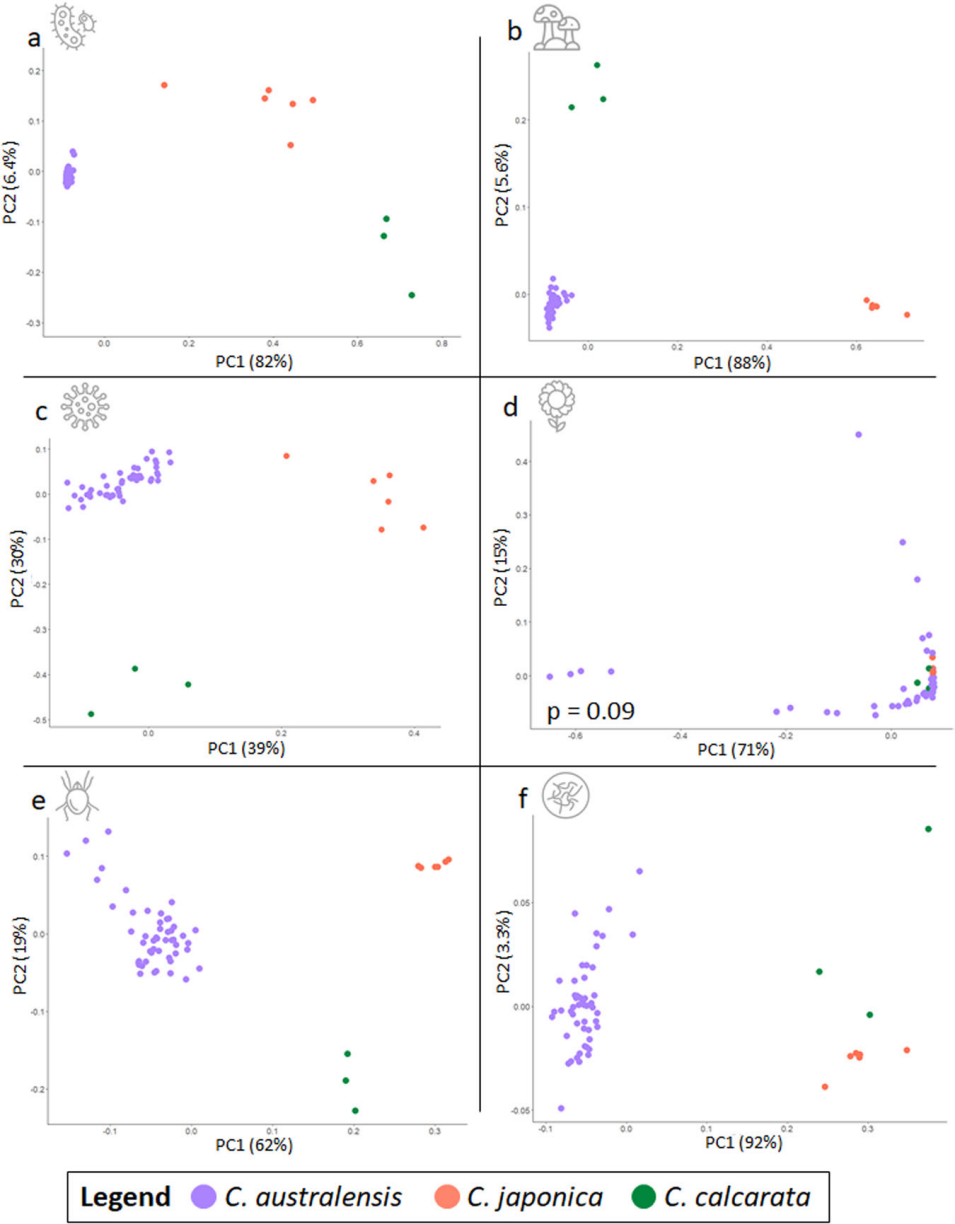
Principal coordinate analyses (PCoA) based on Bray-Curtis dissimilarities between *Ceratina* species *C. australensis*, *C. japonica*, and *C. calcarata*. Plots display relative abundance distributions of family level data from (**a**) Bacteria, (**b**) Fungi, (**c**) Viruses, (**d**) Plants, (**e**) Arachnids, and (**f**) Nematodes. Host species had a highly significant effect on community variance among all phyla (*p* < 0.001) except plants (*p* = 0.09; full results in Data S4).

**Fig. 3:**
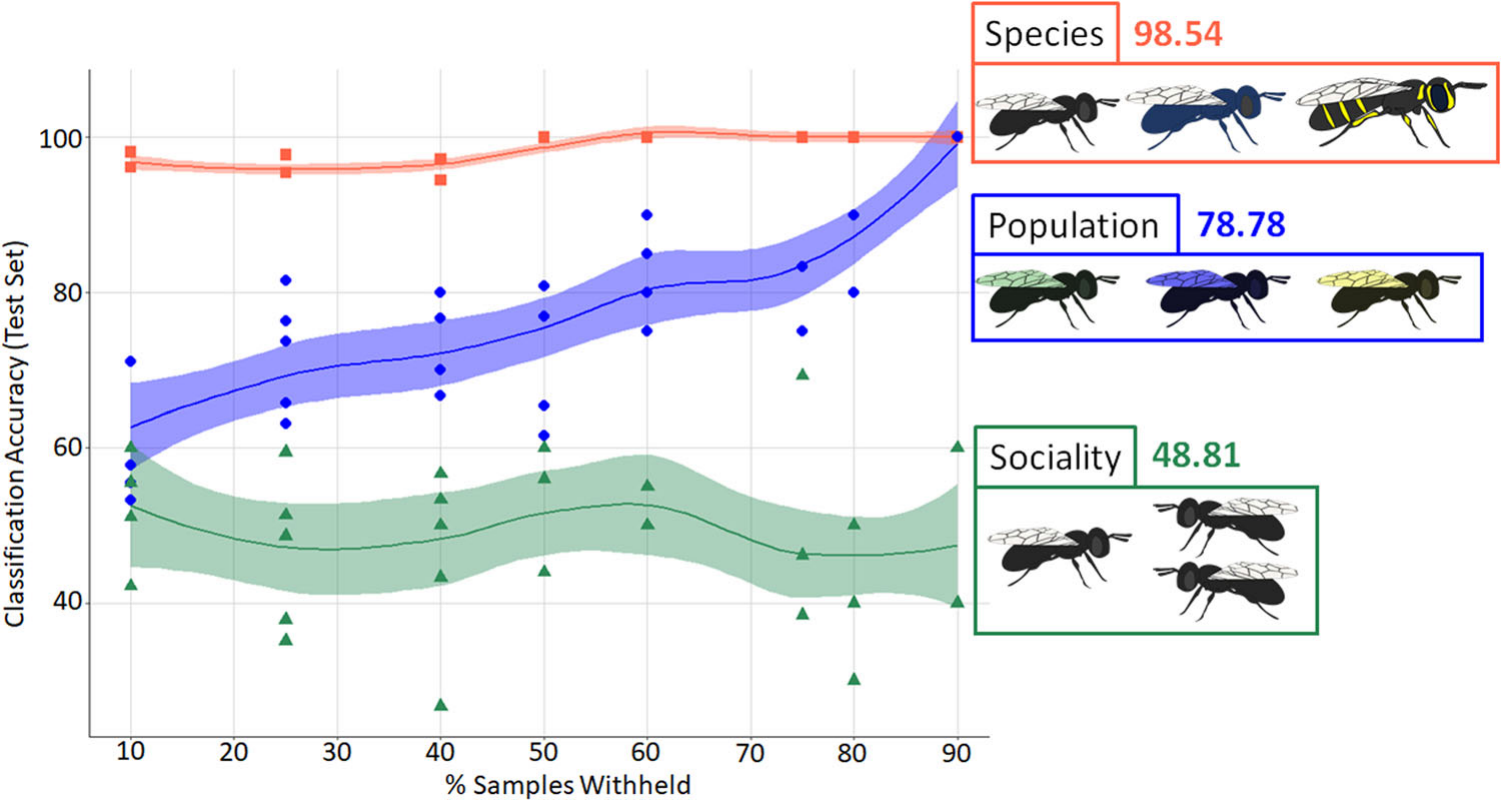
Summary comparison of performance accuracy among three separate random-forest classifiers. Random-forest classifiers were individually trained to assign samples either to species, population of origin, or social phenotype using total metagenomic data and between 10 and 90% of samples for training. Overall average classification accuracy by model is presented. While models trained to assign species or population of origin approached 100% accuracy with increasing training set size, metagenomic data were unable to resolve social phenotype with accuracy better than chance. Full results are in Data S5.

Host species accounted for most of the detectable variation in relative community abundance overall (Figs. 1, 2; PERMANOVA, *R*^2^ ≥ 0.626, *p* < 0.001) and within all assessed groups except plants at both the family and genus levels (e.g., bacterial families, *R*^2^ = 0.8567, *p* < 0.001; Figs. 2a–f, S3, S4; Data S3, S4). Accordingly, a random-forest classifier (RFC) quickly achieved 100% accuracy binning samples by host species (Figs. 3, S12; Data S5) and performed with greater than 95% accuracy even when trained on just 10% of available sample data. Bacteria made up 17 of the 20 most important families for overall RFC model structure (85%), and included some which were also identified during diversity analysis (e.g., Pectobacteriaceae, Data S6, S7). Some of this set also included those families that were significantly overrepresented in one species or another (negative binomial distribution analysis (NBDA), padj <0.05; Fig. S5; Data S8–S10), such as the fungi Wallemiaceae in *C. calcarata* over *C. australensis* and the bacteria Peptococcaceae in *C. australensis* over both *C. calcarata* and *C. japonica* among others. Overall, these WGS-derived metagenomic data reliably and confidently characterized three known host bee species sampled from three highly isolated distributions.

Examining community composition, members of bacteria *Burkholderia*, *Pseudomonas*, *Lactobacillus*, and *Bacillus* were found to be among the largest contributors to variation among all host species (combined average contribution = 22.3%; Data S7). Other notable contributors included the plant families Fabaceae and Chenopodiaceae and viruses Podoviridae and Potyviridae. NBDA identified a total of 286 significantly differentially represented families (DRFs) and 472 differentially represented genera (DRGs) among host bee species (padj < 0.05; Data S8–S10). Notably, at both the family and genus levels, *C. australensis* featured the greatest numbers of significantly overabundant phyla (DRF = 110; DRG = 221) compared to both *C. japonica* (DRF = 30; DRG = 45) and *C. calcarata* (DRF = 22; DRG = 29). Here we focus on results of NBDA at the genus level. Notable significantly overrepresented taxa included the bacterial genus *Lactobacillus* in *C. japonica* and *Burkholderia* in both *C. australensis* and *C. calcarata*; fungal genus *Saccharomyces* in *C. japonica* and *C. australensis*; and bacteria genus *Serratia* in *C. calcarata* and *C. japonica*. Additional taxa of notable abundance included the plant genus *Nicotiana* in *C. australensis*.

Weighted gene co-expression network analysis (WGCNA) corroborated previous analyses and identified a total of 22 modules capturing co-occurrent community members associated with each host *Ceratina* species (Fig. 1; Data S11–S13). This analysis further highlighted modules (i.e., communities) which were highly significantly and positively correlated with each species (i.e., *C. australensis*, cor = 0.99, *p* = 7.2e−139; *C. calcarata*, cor = 0.82, *p* = 1.7e−05; *C. japonica*, cor = 1.0, *p* < 1e−200; Data S14). The most strongly *C. australensis*-associated community was predominantly composed of bacteria (b = 53%) and fungi (f = 22%) with relatively few viruses (v = 8%), and included the Aerococcaceae, Acidobacteriaceae, and Deinococcaceae as hub members. Though *C. japonica*’s key community featured proportionally far fewer bacteria than *C. australensis* or *C. calcarata* overall (b = 9%, f = 27%, v = 13%), hub members included the Cohaesibacteraceae and Helicobacteraceae. Finally, *C. calcarata*’s relatively balanced mix of bacteria, fungi, and viruses (b = 19%, f = 35%, v = 24%) included the Hafniaceae as strongly co-occurrent hub member. Of 19 hub bacterial taxa in the strongly *C. australensis*-associated module, 16 were also found to be among the 50 most important taxa for RFC analysis. These families included Acidobacteriaceae, Borreliaceae, Chromatiaceae, Leptospiraceae, and Spiroplasmataceae (Data S5; S14). Of these, Acidobacteriaceae and Borreliaceae were also both found to be significantly overrepresented in *C. australensis* during NBDA (Data S7).

Despite considerable variation in overall community composition, functional enrichment was highly concordant among host species (Data S15). Bacterial communities in all three bee species were primarily enriched for metabolic (e.g., fructose and mannose metabolism, map00051) and genetic information processing tasks (e.g., protein processing in endoplasmic reticulum, map04141; *N*_concordant_ = 382, 88%; Data S15). Terms uniquely enriched by species included the KEGG pathway response to nicotine (map05033) in *C. australensis*; circadian rhythm (map04710) in both *C. calcarata* and *C. japonica*; and the Toll-like receptor signaling pathway (map04620) in both *C. australensis* and *C. japonica*.

### Regional environment influences diversity of the *C. australensis* microbiome

Core bacterial and fungal groups (defined in this study as >50% prevalence and 1% relative abundance, but see also ref. ^44^) were calculated for each *C. australensis* population of origin (i.e., Queensland, QLD; Victoria, VIC; and South Australia, SA), revealing 13 bacterial and 22 fungal genera present across sampled populations. Core bacterial taxa included *Burkholderia*, *Bacillus*, *Acinetobacter*, and *Paenibacillus*; core fungi included *Aspergillus*, *Saccharomyces*, and *Penicillium* (Data S16). These core genera were identified within a total of 383 families (75% of 509 families total) present in all three host *C. australensis* populations. Other notable taxa identified across populations included several potential plant pathogenic virus families Betaflexiviridae, Luteoviridae, Partitiviridae, Potyviridae, Secoviridae; with families Bromoviridae and Virgaviridae additionally detected in VIC and QLD (Data S17).

**Fig. 4:**
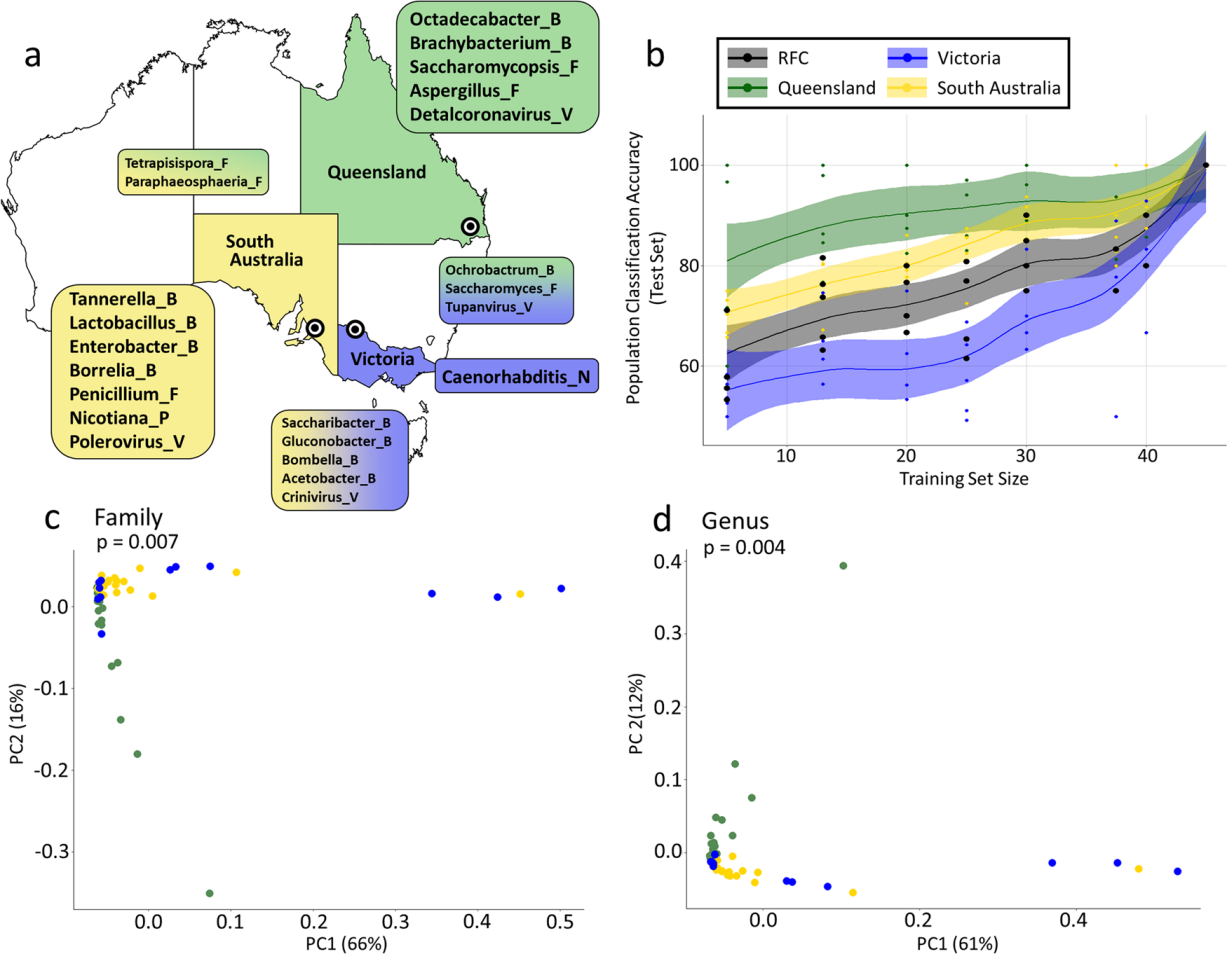
Microorganismal community composition by *C. australensis* population. **a** A selection of highly differentially abundant genera (Log2FC > 2.0) detected among sampled populations. Bacterial families accounted for the greatest number of highly abundant taxa cross populations. **b** Classification accuracy of a random-forest model trained to assign population of origin to samples as a function of the size of the training set. All trials performed with significantly greater accuracy than would an estimated random draw (*p* < 0.05). While total model accuracy approached 100% with inclusion of 40+ samples, performance was consistently better for Queensland and South Australia compared to Victoria. c and d) PCoA plots are shown for total metagenomic abundance data analyzed by family (**c**) and genus (**d**) across *C. australensis* populations of origin. Ordination was comparable at both levels of resolution; and PERMANOVA revealed that overall community composition was significantly distinguished by population of origin; greatest differences were between Queensland and both South Australia and Victoria.

**Fig. 5:**
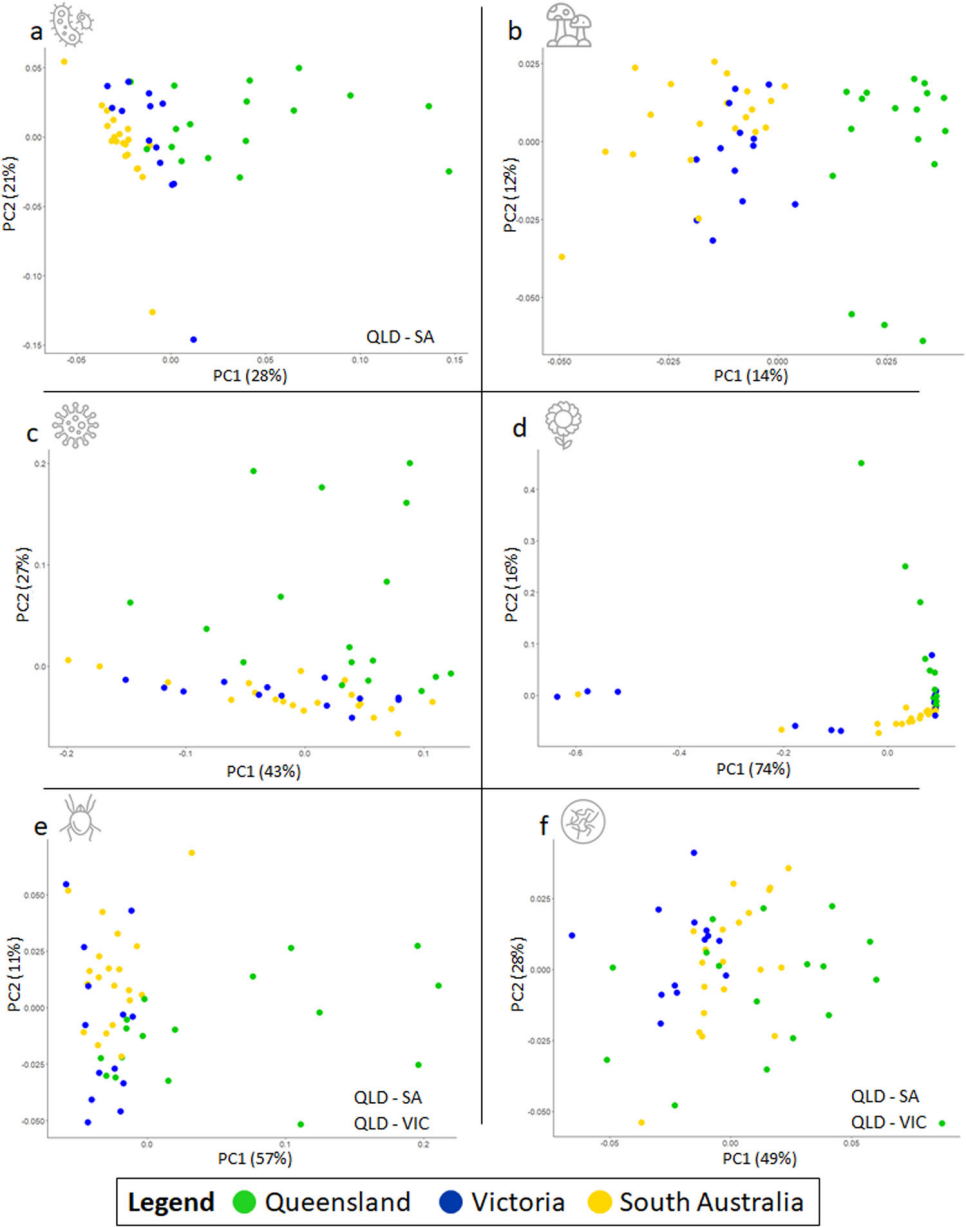
Principal coordinate analyses (PCoA) based on Bray-Curtis dissimilarities between three focal *Ceratina australensis* populations: Queensland (QLD), Victoria (Victoria), and South Australia (SA). Plots display relative abundance distributions of family level data from (**a**) Bacteria, (**b**) Fungi, (**c**) Viruses, (**d**) Plants, (**e**) Arachnids, and (**f**) Nematodes. Population of origin had a highly significant effect on community variance among all phyla (*p* < 0.001); in cases of heterogeneous dispersion, significantly distinct populations are indicated (**a**, **e**, **f**; full results in Data S18).

Populations had significant differences in overall community composition (PERMANOVA, *R*^2^ = 0.148, *p* = 0.007; Fig. 4c, d; Data S18) and among phyla (PERMANOVA, p ≤ 0.005; Figs. 5, S6, S7). Post hoc testing revealed significant differences in community composition specifically between QLD and SA (Tukey, padj = 0.00041) as well as QLD and VIC (Tukey, padj = 0.0323; Fig. 5e; Data S18). RFC corroborated multivariate analyses, which supported QLD as the most distinct metagenomic population, followed by SA, and then VIC (Figs. 4b, S12; Data S5, S6). The most informative families for RFC accuracy included bacteria (e.g., Clostridiaceae, Cellulomonadaceae), fungi (e.g., Agaricaceae, Hypocreaceae), and viruses (Mimiviridae, Peribunyaviridae), some of which were also identified during diversity analysis (e.g., Clostridiaceae; Mimiviridae; Data S5; S19). Of these highly informative families, the Clostridiaceae and Agaricaceae were found to be overrepresented in QLD and VIC compared to SA; the Mimiviridae and Peribunyaviridae in QLD compared to both VIC and SA; and the Hypocreaceae in SA compared to QLD (Data S20).

Each population had distinct taxonomic diversity and community composition (Data S19). Members of bacterial genus *Burkholderia*, accounted for the greatest percentage of dissimilarity among any two populations (~5%), with slightly higher average abundances in SA over VIC and VIC over QLD. NBDA corroborated diversity analyses and identified a total of 153 significantly DRFs and 191 DRGs among *C. australensis* populations (adjusted *p* < 0.05; Data S20, S21) with a majority of uniquely overrepresented taxa identified in QLD (DAF = 57; DAG = 62) followed by SA (DAF = 37; DAG = 58) and VIC (DAF = 17; DAG = 14). These results also corroborate RFC performance by reinforcing that QLD may be the most distinct of the three populations. By contrast, SA featured more than twice as many strongly overrepresented genera as QLD or VIC (*N*_log2FC>2_ = 78, Fig. 4a), including *Lactobacillus*, *Nicotiana*, and *Polerovirus*.

Although there were some notable variations in community composition across the three *C. australensis* populations, functional enrichment was again largely concordant across locations (Data S22). All three populations shared KEGG enrichment for metabolic processes (e.g., fructose, map00051; tyrosine, map00350), environmental information processing (e.g., quorum sensing, map02024), and protein signaling (e.g., oxidative phosphorylation, map00190; Data S22). Unique KEGG pathway enrichment for each population included B-cell receptor signaling (map04662) in QLD, nicotine addiction (map05033) in SA, and Toll-like receptor signaling (map04620) in VIC.

### Metagenomic signals of sociality in *C. australensis*

Comparisons of solitary to social nests across populations revealed no universal effect of *C. australensis* sociality on metagenomic diversity (e.g., bacteria; PERMANOVA, *R*^2^ = 0.022, *p* = 0.379; Figs. S8, S9; Data S23) or relative abundance across populations (Data S24, S25). Reassessing the effects of sociality *within* each population revealed evidence of significant variations in diversity and abundance at both the family and genus levels across all phyla (Figs. S10, S11; Data S26–S28), including overrepresentation of *Acetobacter* in solitary over social hosts (Log2FC = 37.18, *p* = 6.35E−06) and both *Streptococcus* and *Fusobacterium* in social over solitary in the Victoria population (Data S28). These differences in overall community composition were not uniform, however; the bacterial genera *Bombella* and *Gluconobacter*, for example, were overrepresented in the social hosts of SA but also in the solitary nest individuals of VIC (Data S28). Lastly, a RFC was unable to ever perform significantly better than a simulated random draw, and failed to surpass 50% accuracy in assigning samples to their correct social or solitary bins across all populations (Figs. 3, S12**;** Data S5).

### Core wild bee microbiomes in comparison with corbiculates

**Fig. 6:**
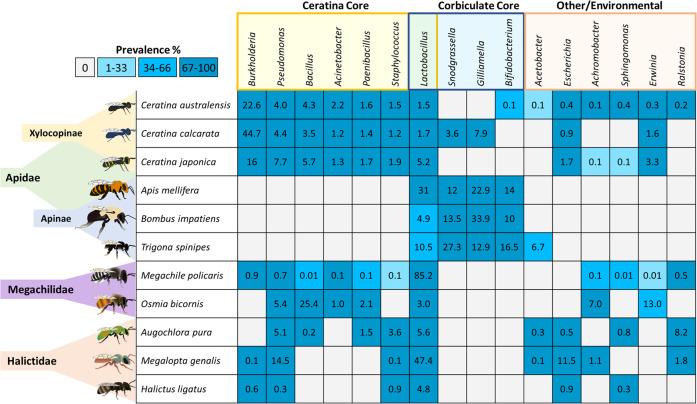
Highlighted selection of some of the most prevalent and abundant bacterial genera identified among bee lineages. The *Ceratina* core microbial genera (>50% prevalence and >1% relative abundance) are also found in other solitary and socially polymorphic bees. The previously identified corbiculate core microbiome is largely limited to that group. Other key bacterial genera may have their origins in the environment, and may be commensal in lineages with less well-established intranidal environments. Only the highly diverse *Lactobacillus* genus has been identified in almost all bee lineages studied to date (full dataset for all species can be found in Data S30).

Comparison of bacterial community composition among 38 bee species (Data S29, S30) revealed greater overall similarity between the *Ceratina* (Xylocopinae) and either the leafcutter (Megachilidae) or sweat bees (Halictidae) than with corbiculate bees from their sister subfamily Apinae (Fig. 6). Although the *Ceratina* are collectively host to a richly diverse suite of bacteria, their core microbial community is primarily composed of *Burkholderia* (average relative abundance = 22.3%), *Pseudomonas* (4.6%), *Bacillus* (4.5%), and *Acinetobacter* (2.1%). Genera from this set, such as the *Pseudomonas*, are effectively undetected among corbiculates, but occur with high relative abundance among both the Megachilidae (e.g., *Osmia bicornis*, 5.4%) and Halictidae (e.g., *Augochlora pura*, 5.1% and *Megalopta genalis*, 14.5%). Other members of the *Ceratina* microbiome such as *Escherichia* and *Erwinia*, which represent prevalent but low abundance organisms, were also detected in the Megachilidae and Halictidae. Only the highly diverse bacterial genus *Lactobacillus* was detected with relatively high prevalence and abundance across all major bee lineages and in every host species considered with notable exceptions in the oil digger bees (*Centris atripes* and *Anthophora abrupta*) and the stingless bee, *Tetragonula fuscobalteata* (^20^ Data S29, S30).

## Discussion

Here we present a first comparative metagenomic analysis of three wild bee species drawn from three continents—Australia, North America, and Asia. We consider the implications of variations in microbiome among host species, investigate how differences in *C. australensis* population of origin may reflect on species ecology and sociobiology across its range in Australia, and compare the first-ever core microbiome among the carpenter bees to similar datasets among other bee lineages.

### The wild bee microbiome is diverse and unique to host species

Carpenter bee host species had distinct metagenomic signatures which were clearly detectable by multivariate, clustering, and machine learning algorithms. Most notably, RFC resolved host species with >95% accuracy even when trained on just 10% of available data, suggesting a very clear and strong signal in taxonomic diversity and abundance data. A total of 43 of the top 50 most informative taxa for host bee species classification accuracy were bacterial families including the Acetobacteraceae and Acidobacteriaceae. Acidobacteriaceae was additionally found to be a hub taxon within the strongly *C. australensis*-associated module of co-occurrent taxa, and significantly overrepresented in *C. australensis* compared to both *C. japonica* and *C. calcarata*. These results collectively suggest that members of Acidobacteriaceae may feature centrally in *C. australensis* biology. Both Acetobacteraceae and Acidobacteriaceae contain acidophilic genera and have been detected in other bees as possible mutualists taken up from the environment, often through pollen provisions^7^. Although the composition, nutritional value, and microbial associations of pollen provisions have been assessed among *Ceratina*^38,39,45^, including *C. australensis*^41^, additional investigations could offer finer scale insights into the combined influences of floral resources and intranidal conditions on microbiome composition and fitness in this group.

Notably, all of *C. australensis*’s hub taxa were bacterial families and included those found to play important roles in other bee lineages (e.g., Prevotellaceae in *Apis florea*;^46^ Caulobacteraceae in *Osmia*;^47^). By contrast, only two of *C. japonica*’s 14 hub taxa were bacterial families (Cohaesibacteraceae and Helicobacteraceae), with the remainder including wood-rotting fungal families (Grifolaceae and Punctulariaceae) and plant viral families (e.g., Tospoviridae); and similarly, of *C. calcarata*’s five hub taxa, Hafniaceae was the only bacterial family. Although each of these hub taxa were also found to be significantly overrepresented in their host species, future studies with extended sampling of *C. japonica* or *C. calcarata* would better clarify the degree to which these families play roles of considerable biological importance to their hosts.

Despite considerable variation by host species, functional enrichment across our three *Ceratina* species was found to be largely uniform, arguably highlighting a core profile of microbial activity. In general, bacterial families detected in each of our *Ceratina* hosts likely contribute to some combination of improving nutrient uptake for their hosts (e.g., via carbohydrate metabolism) and performing tasks associated with immune response (e.g., Toll signaling pathway; T- and B-cell receptor signaling). There is evidence, primarily in honey bees, that gut microbes may strongly aid in host immune and metabolic pathways^15,16,18^. Our results reinforce previous assessments in suggesting that the microbiomes of solitary bees may also contribute to essential biological processes for their hosts, regardless of variations in ecology, climate, or even overarching community composition^47,48^. Future studies that incorporate RNAseq methods alongside metagenomic analyses should be able to further tease apart variations in microbial diversity and function among host bee species.

### Wild bee microbiomes may be strongly influenced by local environment

We found that the *C. australensis* microbiome varies significantly across its range, most likely attributable to both physical isolation and ecological variation. In our study, Queensland not only featured the most unique and significantly overrepresented taxa but also the most distinct metagenomic profile across all analyses. Our metagenomic data are thus broadly consistent with former amplicon, microsatellite, and genome-wide datasets used in previous *C. australensis* studies (e.g.,^40,41,43^.) which found similar dimensions of structure among these populations. Gradually dispersing from an origin population in Queensland^49^, *C. australensis* is thought to have spread south and west following the Murray River to reach its current distribution^40^. This trajectory suggests that any substantial variation in metagenomic profiles between Queensland and either South Australia or Victoria should reveal relatively novel elements associated with those environments. Comparing variation in microbiomes by population, the bacterial genus *Pantoea* was found uniquely in the Queensland core, *Streptococcus* uniquely in the Victoria core, and both *Flavobacterium* and *Desulfovibrio* uniquely within the South Australia core. *Pantoea* is a highly diverse genus and well-studied plant pathogen which can form mutualistic and even commensal associations with insect hosts (e.g., leafcutter ants,^50,51^. Further, members of *Pantoea* may be readily vectored among plant hosts by honey bees^52^ and have even been identified in the guts of Australian stingless bees (Apidae: Meliponini) sampled in Queensland^53^. Detection of *Pantoea* in the Queensland *C. australensis* core suggests it may also be vectored by this species. *Streptococcus* bacteria are a widely pathogenic group, well-studied in honey bees^54^ and known in Australian honey bee populations^55^. Widespread detection of *Streptococcus* and *Enterococcus* (formerly classified as *Streptococcus*), especially in Victorian *C. australensis*, may indicate a pervasive—if ultimately non-lethal—bacterial challenge for that population. Finally, members of *Flavobacterium*^56^ and *Desulfovibrio*^57^ are both known to favor saline, marine environments. Although their biological role in *C. australensis* remains unknown, their strong representation in the South Australian population—sampled exclusively from beach dunes along the Great Australian Bight—helps to illustrate the degree to which the *C. australensis* microbiome may be directly influenced by regional environment.

Turning to insights regarding *C. australensis*’s diet, although all populations appear to be closely associated with *Solanum* (nightshades), *Vigna* (legumes), and *Gossypium* (mallows, including cotton), we did find evidence in support of previous studies^41^ that pollen usage varies considerably across *C. australensis*’s range. For example, though South Australian *C. australensis* include more *Brassica* (canola and mustard) and *Chenopodium* (goosefoots) than Queensland, northeastern bees are associated with more *Medicago* (legumes), *Glycine* (soybeans), and *Sorghum* (cereals). Functional enrichment offers some insights into floral resources among populations, such as our detecting evidence of nicotine among *C. australensis* in South Australia. Secondary floral compounds such as nicotine or other plant alkaloids can strongly influence foraging preferences in honey bees^58^, bumble bees^59^, and among other wild bee species^60^. Although the degree to which such compounds might similarly affect *Ceratina* remains an open question, plant genus *Nicotiana* was accordingly detected among South Australian *C. australensis* significantly more than in Queensland. Notably, the distribution of several known Australian members of *Nicotiana*, including wild tobacco, overlaps closely with that of *C. australensis* in both South Australia and Victoria^61^.

Unique variations aside, we found that all three *C. australensis* populations share most of their core bacterial and fungal genera, a result which echoes previous microbial metabarcoding studies^38^. Of special note, bacterial genus *Burkholderia* featured prominently as the most abundant group of each population. *Burkholderia* are distinguished as one of the most common environmental bacterial genera^62^, and its members are known to have an expanded range across Australia^63^. As discussed further below, *Burkholderia* are known to form mutualistic relationships with some insect hosts^64^, but it remains unclear what their role may be in *C. australensis* and among the *Ceratina* globally. Among the best represented core fungal genera were *Aspergillus*, *Fusarium* and *Saccharomyces*. Depending on species, members of each of these fungal genera may be helpful or harmful as reported from honey bee hosts in which they have primarily been studied to date (e.g.,^65–67^.). This is the first detection of these genera in *C. australensis*^41^, and an investigation into whether they may play a commensal or harmful role across its populations, particularly during a time of dynamic climatic change^68^, remains an important target for future studies.

These data also provide preliminary assessments into the degree to which *C. australensis* may be vectoring plant diseases in its environment, including those which may be harmful to economically valuable crop species (e.g., plum pox and *Prunus*^69^,). We identified seven major plant viral families among our sampled *C. australensis* populations that had been previously found only in Australian honey bee populations^70^. Our dataset of viral families—which includes the Secoviridae, Potyviridae, and Luteviridae among others—also suggests these groups may be more widespread than detected by Roberts^70^. Intriguingly, abundances of these viral families follow similar trends over the landscape in both studies where direct comparisons between sampling areas could be made, with overall highest loads identified among South Australian bees, and lowest among those from Queensland. Notably, both studies also found evidence of Virgiviridae: *Tobamovirus*, a genus of the very recently established cucumber green mottle mosaic virus, primarily in Queensland.

Metabarcoding studies suggest that the diets of both introduced honey bees and Australia’s wild bee species may overlap considerably^71^, potentially branching into crop variety. The use of even managed bees as a means of plant pathogen surveillance is of great value, but still very much in its infancy^70,72^. To the best of our knowledge, our study represents a critical first demonstration that wild bees can also offer invaluable insights into the spread of both established and newly introduced crop diseases. Metagenomic screening of *C. australensis* and other wild bee populations, which benefit from robust and ever improving reference datasets on NCBI, thus offers ecologically comprehensive insights into both managed and unmanaged landscapes in Australia and elsewhere.

### There are detectable metagenomic signals of sociality within *C. australensis* populations

Previous research has shown that around 13% of *C. australensis* females consistently demonstrate cooperative breeding across populations in the species, a strategy which is thought to be advantageous under heavy parasite pressure^42,73^. Metagenomic data indicate that *C. australensis* populations do experience significantly variable environmental factors across their distribution and, intriguingly, the metagenomes of solitary and social individuals appear to differ significantly within populations. For example, among South Australian *C. australensis*, the bacterial family Thermomonosporaceae is very well represented in solitary bees, while both Brucellaceae and Prolixibacteraceae are found in social bees. These data suggest there is significant co-variation between bacterial community and host bee sociality. A recent genome-wide association study in *C. australensis* found that many genetic loci may be tied to social phenotypes, but each locus may have only a relatively small influence^43^. Signatures of selection by sociality within each *C. australensis* population detected in that study are concordant with our metagenomic results overall, suggesting wild bees more strongly experience population-level rather than species-level differentiation among social forms^43,74^.

### Social complexity is inversely proportional to microbiome diversity across bees

The carpenter bee core bacterial microbiome presented here represents a first metagenomic profile for non-corbiculate wild bees, a valuable point of comparison outside of the more socially complex bees (i.e., corbiculates) in which most metagenomic and microbiological work has been done to date^20,75,76^. The carpenter bee core is comprised of 11 bacterial genera, predominantly *Burkholderia* (22.3%) followed by *Pseudomonas* (4.6%) and *Bacillus* (4.5%); all three genera play important roles across insects^64,77^. In particular, members of the *Burkholderia* have been found to act as symbionts for a variety of insect hosts in which they have been studied (e.g., the bean bug, *Riptortus pedestris*^78^), performing a wide suite of beneficial functions (e.g., nutritional supplementation;^64^). Among bees, *Burkholderia* and *Pseudomonas* are thought to be acquired from the environment (e.g., soils and plants) and have been detected primarily among solitary species;^7,9,79^ though *Pseudomonas* may occasionally be present in some corbiculates^80,81^. Accordingly, we found that the largely solitary carpenter bee core shares almost no microbial members with their highly social sister corbiculate bees from the subfamily Apinae, which features a relatively small core set (Fig. 6). Unlike the corbiculate bees, the nest structure, colony size and social environments of the Xylocopinae, Megachilidae, and Halictidae are relatively small, solitary and exposed to the environment (e.g., soil or decaying branches). Observed consistencies in the composition of small carpenter, leafcutter, and sweat bee microbiomes therefore appears to highlight the importance of the physical rather than social environments in establishing the microbial communities of those host species^48,82^. *Lactobacillus* was the only bacterial genus from the carpenter bee core to be detected in all bee species measured, often at comparatively high relative abundances. The *Lactobacillus* genus is highly diverse, and its members have established as symbionts across a similarly diverse range of both vertebrate^83,84^ and invertebrate hosts^85^. *Lactobacillus* are known to be highly beneficial in honey bees, in which they have been extensively studied, often offsetting the deleterious effects of honey bee diseases like chalkbrood^86,87^. Species and strains of *Lactobacillus* very likely play similarly commensal roles in other bee hosts (e.g., Megachilidae^88^; *Nomia melanderi*^82^) and, alongside many other microbes, are critical to the diet, fitness and health of many bees^13,89^.

Previous research suggests that even distantly related bee lineages experience similar evolutionary dynamics as a product of their social complexity^37,74^. Although microbial composition may be closely tied to host species, lineage sociality and individual behavioral caste also appear to play critical roles in shaping community structure^20,67^. The degree to which there may be any consistent bidirectional influence between microbial community composition and species sociality across major bee lineages remains an open question for future hologenomic research. For example, do the microbiomes of obligately eusocial bees outside of Apinae (e.g., *Exoneurella tridentata*^90^) more closely resemble those of other members of their subfamily (Xylcopinae) or those of other eusocial bees (e.g., Apinae) despite phylogenetic distance? Future studies which sample microbiomes from consistent life stages and tissue types across a comparably wide range of bee families may yield refined insights. We are on the cusp of tackling these and other questions as metagenomic methods continue to improve^91^ and comparable metagenomic studies are performed in bees across independent origins of sociality^92,93^. These works promise to provide additional datasets that will be invaluable for further illuminating comparisons and that should contribute to a progressively well-defined spectrum of environmental through intranidal origins of bee metagenomes.

## Methods

### Sampling and sequencing

We collected a total of 60 bee samples over 3 years from three species of *Ceratina*. The sample set of 51 adult female *Ceratina australensis* were collected in January 2016 from three distinct populations in Australia: Queensland (N = 18; 28.24°S, 152.09°E), Victoria (N = 13; 34.15°S, 142.16°E), and South Australia (N = 20; 34.94°S, 138.50°E). Six females of *C. japonica* were collected in Sapporo, Japan (N = 6; 43.06°N, 141.35°E) in July 2015, and three females of *C. calcarata* were collected in Durham, New Hampshire in July 2017 (N = 3; 43.14°N, 70.94°W). All bees were flash frozen in liquid nitrogen during collection to preserve DNA integrity prior to whole body genomic DNA extraction via phenol-chloroform protocol^94^. Samples were then submitted to Genome Quebec for PCR-free library construction (NEB Ultra II kit) and Illumina HiSeq 2500 (125 PE) sequencing at an average depth of 30M reads per sample.

### Sequence data processing

Raw Illumina reads of whole-genome data from *C. australensis*, *C. japonica*, and *C. calcarata* were quality checked with *FastQC* (https://www.bioinformatics.babraham.ac.uk/projects/fastqc/). Adapters were removed and reads were cleaned with *Trimmomatic*^95^, with the following settings: TRAILING:20 SLIDINGWINDOW:4:20 MINLEN:36. The cleaned read pairs were mapped to the appropriate reference genome for each species with *bwa mem* using default settings^96^, and unmapped reads were extracted from the resulting bam files with samtools (^97^ Data S1). An average of 93% of *C. australensis* reads mapped to the *C. australensis* reference genome (NCBI: ASM430768v1;^35^); 96% of *C. calcarata* reads mapped to the *C. calcarata* reference genome (NCBI: ASM165200v1;^34^); and 87% of *C. japonica* reads mapped to the *C. japonica* reference genome (NCBI PRJNA413373;^37^). Reads were considered unmapped if at least one read from the pair did not map to the reference genome; and paired unmapped reads, in fastq format, were used for further analysis. All sequencing data generated for this study can be accessed via NCBI PRJNA407923 and were handled as compositional data during analysis^98^.

### Taxonomic classification

Taxonomic classification of reads unmapped to the reference genome was carried out with the use of Kraken2 software^99^. Paired reads were classified with default settings against the latest available *nt*—NCBI non-redundant nucleotide database. Read counts were used to calculate relative abundance at the family level separately for each of six major biological groups, including: bacteria, fungi, plants, arachnids, nematodes, and viruses. Relative abundance of each family was calculated by dividing number of reads classified to family by the total number of reads classified to a particular group among samples. We then repeated this analysis at the level of genus.

We tested several filtering strategies to ensure low rate of false positive classifications without substantial loss of information. The performance of the following thresholds was assessed: (i) 1% minimum relative abundance within a given sample, (ii) 0.1% minimum relative abundance within a sample, (iii) 0.1% minimum average relative abundance across all samples per species, (iv) minimum number of 10 classified reads (Fig. S1). To explore the effect of the library size on classified taxa and their relative abundance we performed the above-described taxonomic classification for several levels of input reads subsampling, including 0.05–1.25 M paired reads, to construct the rarefaction curve (Fig. S2). All samples were found to be within the rarefied range, and a filter threshold of 0.1% was selected for further analyses.

We also performed taxonomic classification using an assembly-based approach. First, we merged the reads from all *C. australensis* individuals. Then, we ran the assembly using metaSPAdes^100^ with paired-end library type and default settings, including k: 21, 33, 55. We then excluded contigs shorter than 300 bp, and classified those remaining based on best BLAST hits^101^ to the nucleotide (*nt*) database. We then used *bwa mem* with default settings to map the reads from each sample against the assembly contigs. We recovered the mapped reads with samtools and assigned each to the taxonomic classification of the contig to which they mapped. Both metaSPAdes and Kraken2 taxonomic assignments were largely concordant with results of BLASTn runs (using default parameters, with max target seqs set to 1 and minimum shared ID set to 70%; Table S2), so we used Kraken2 assignments for further analyses.

### Taxonomic diversity and dissimilarity analyses

Bray-Curtis dissimilarity matrices were calculated for each taxonomic group and assessed via principal coordinates analysis (PCoA) and non-metric multi-dimensional scaling (NMDS) analysis in the R package Vegan^102^. We applied PCoA and associated analyses in this instance as these tests are better reveal qualitative differences among sets, rather than a principal components analysis which better assesses quantitative similarities. To test whether species or population of origin had a significant effect on variation in community composition we then performed permutational multivariate analyses (PERMANOVA) via the *ADONIS* function in Vegan. To ensure that the assumptions of homogeneity of group dispersions was met, we ran *BETADISPER* and assessed the resulting distances to the group centroids via *ANOVA* followed by post hoc Tukey’s range test to determine whether there were significant pairwise differences among groups. Next, we performed similarity percentage (SIMPER) analyses within PAST v 4.06^103^ to identify which taxa were primarily responsible for observed differences between groups.

### Functional analysis

We performed functional analysis of reads classified as of bacterial or fungal origin by the taxid information in the Kraken2 classification output. These were then aligned to the *nr*—NCBI non-redundant protein database using DIAMOND^104^ with default fast mode, filtering for e-value of 1e−10 and returning maximum five alignments per read. As DIAMOND does not support paired-end mode we performed functional analysis based on forward reads only. We checked taxonomy of the resulting alignments and excluded from further analyses reads that did not have bacterial or fungal hits among the five alignments. Then we used FragGeneScan^105^ to find genes in the reads, and analyzed the resulting amino acid fasta files with GhostKOALA^106^ and eggNOG-mapper^107^ to characterize gene functions based on KEGG identifiers and the reconstructed pathways.

### Negative binomial distribution analysis  + weighted gene co-expression network analysis

As usefully applied in other recent metagenomic works (e.g., Rothman et al.^17^; Kapheim et al.^82^), significantly differentially abundant phyla were characterized using NBDA to determine DRFs and differentially represented genera (DRGs) (DESeq2^108^), comparing reads by *Ceratina* species (i.e., *C. japonica* vs. *C. calcarata* vs. *C. australensis*), *C. australensis* populations (i.e., Queensland vs. Victoria vs. South Australia), and *C. australensis* social phenotype across (i.e., solitary vs. social) and within populations (e.g., Queensland social vs. Queensland solitary). We then used weighted gene co-expression network analysis (WGCNA^109^) to further assess microbiome communality (i.e., co-occurrence) among *Ceratina* species. WGCNA is a powerful network analysis option, and one which lends itself to illuminating visualization for figures downstream. Following standard protocol to prepare for this analysis^110^, normalized read data were filtered to remove any taxa that featured too few or no read values in at least one sample set, and any samples that appeared as clear outliers following hierarchical clustering. This preparatory step indicated relatively weak distinctions among both population and sociality data, so only analysis by host species was completed. Sample SJ19 was removed as a group outlier, and samples were then assigned biological trait data (i.e., host species). We selected a soft power of 6, which indicated explanation of well over 90% of the data, and performed the remainder of network analysis following standard protocols. Taxa which were assigned both trait significance and module membership values >0.90 were considered “Hub” taxa, of especially high association with host species and high co-occurrence among other members of their module (i.e., community).

### Random-forest classifiers

We first trained three individual RFCs in R (package randomForest^111^) to assess the degree to which metagenomic communities effectively predicted *Ceratina* host species, and population of origin and sociality across populations within *C. australensis*. We then trained a fourth RFC to assess sociality as partitioned by population (6 bins) and then established three additional RFCs to test sociality within each population (2 bins per RFC). We trained each RFC using total metagenomic read data (i.e., all phyla) and eight training set sizes (between 10 and 90% of available samples). We then evaluated classification accuracy on the withheld 90 to 10% remaining samples five times a piece, for a total of forty trials per RFC. Prior to each run, we applied the tuneRF function to determine an mtry value (used to facilitate forest creation during the RFC run) which would minimize error. R package caret^112^ was then used to produce a confusion matrix to assess overall RFC performance in accuracy, sensitivity, and specificity, as well as statistical support. As models of significant and robust accuracy (e.g., >80%) featured high average specificity and sensitivity scores (e.g., >80%) we focused on reporting overall accuracy and significance in the main text. Post hoc analyses were performed using the R package RandomForestExplainer^113^ to evaluate the overall degree to which each taxon influenced RFC model accuracy (i.e., individual importance).

### Comparative analysis

To determine the variation in microbial community composition across bee lineages, we first characterized the core bacterial community of our three *Ceratina* species as those genera which were present in more than 50% of samples and featured an average relative abundance >1%^14^. We then compared the composition and relative abundances of all bacterial genera detected among our *Ceratina* species to similar datasets from 35 additional bee species (Data S29, S30). Prior to comparison, we consolidated bacterial composition and count data from these additional datasets to the level of genus before recalculating relative abundance and prevalence.

### Reporting summary

Further information on research design is available in the Nature Research Reporting Summary linked to this article.

## Supplementary information

Supplementary Information

Description of Additional Supplementary Files

Data S1-S30

Reporting Summary

## Data Availability

All newly generated metagenomic data used in this study can be freely accessed via NCBI BioProject number PRJNA407923.

## References

[1] Engel MS (2000). A new interpretation of the oldest fossil bee (Hymenoptera: Apidae). Am. Mus. Novit..

[2] Michener, C. D. *The Bees of the World* 2nd edn, (John Hopkins University Press, 2007).

[3] Klein AM (2007). Importance of pollinators in changing landscapes for world crops. Proc. R. Soc. B..

[4] Fürst M, McMahon DP, Osborne JL, Paxton RJ, Brown MJF (2014). Disease associations between honeybees and bumblebees as a threat to wild pollinators. Nature.

[5] McMahon DP, Wilfert L, Paxton RJ, Brown MJF (2015). Emerging viruses in bees: from molecules to ecology. Adv. Virus Res.

[6] Koch H, Abrol DP, Li J, Schmid-Hempel P (2013). Diversity of evolutionary patterns of bacterial gut associates of corbiculate bees. Mol. Ecol..

[7] McFrederick QS (2012). Environment or kin: whence do bees obtain acidophilic bacteria?. Mol. Ecol..

[8] McFrederick QS, Wcislo WT, Hout MC, Mueller UG (2014). Host species and developmental stage, but not host social structure, affects bacterial community structure in social polymorphic bees. FEMS Microbiol. Ecol..

[9] McFrederick QS (2017). Flowers and wild megachilid bees share microbes. Microb. Ecol..

[10] Jones JC (2018). The gut microbiome is associated with behavioural task in honey bees. Insectes Sociaux.

[11] Kristensen, T. N., Schonherz, A., Rohde, P. D., Sorensen, J. G. & Loeschcke, V. Strong experimental support for the hologenome hypothesis revealed from *Drosophila melanogaster* selection lines. *bioRxiv*10.1101/2021.09.09.459587 (2021)

[12] Bovo S, Utzeri VJ, Ribani A, Cabbri R, Fontanesi L (2020). Shotgun sequencing of honey DNA can describe honey bee derived environmental signatures and the honey bee hologenome complexity. Sci. Rep..

[13] Dharampal PS, Carlson C, Currie CR, Steffan SA (2019). Pollen-borne microbes shape bee fitness. Proc. R. Soc. B..

[14] Graystock P, Rehan SM, McFrederick QS (2017). Hunting for healthy microbiomes: determining the core microbiomes of *Ceratina, Megalopta*, and *Apis* bees and how they associate with microbes in bee collected pollen. Conserv. Genet..

[15] Engel P (2016). The bee microbiome: impact on bee health and model for evolution and ecology of host-microbe interactions. MBio.

[16] Voulgari-Kokota A, McFrederick QS, Steffan-Dewenter I, Keller A (2019). Drivers, diversity, and functions of the solitary-bee microbiota. Trends Microbiol.

[17] Rothman JA, Leger L, Graystock P, Russell K, McFrederick QS (2019). The bumble bee microbiome increases survival of bees exposed to selenate toxicity. Environ. Microbiol..

[18] Engel P, Martinson VG, Moran NA (2012). Functional diversity within the simple gut microbiota of the honey bee. PNAS.

[19] Engel P, Moran NA (2013). Functional and evolutionary insights into the simple yet specific gut microbiota of the honey bee from metagenomic analysis. Gut Microbes.

[20] Kwong WK (2017). Dynamic microbiome evolution in social bees. Sci. Adv..

[21] Breeze TD, Bailey AP, Balcombe KG, Potts SG (2011). Pollination services in the UK: How important are honeybees?. Agric. Ecosyst. Environ..

[22] Dharampal PS, Hetherington MC, Steffan SA (2020). Microbes make the meal: oligolectic bees require microbes within their host pollen to thrive. Ecol. Entomol..

[23] Keller A (2021). (More than) hitchhikers through the network: the shared microbiome of bees and flowers. Curr. Opin. Insect.

[24] Hugenholtz P, Tyson GW (2008). Metagenomics. Nature.

[25] Galbraith DA (2018). Investigating the viral ecology of global bee communities with high- throughput metagenomics. Sci. Rep..

[26] Regan T (2018). Characterisation of the British honey bee metagenome. Nat. Commun..

[27] Bovo S (2018). Shotgun metagenomics of honey DNA: Evaluation of a methodological approach to describe a multi-kingdom honey bee derived environmental DNA signature. PLOS ONE.

[28] Schoonvaere K (2016). Unbiased RNA shotgun metagenomics in social and solitary wild bees detects associations with eukaryote parasites and new viruses. PLOS ONE.

[29] Cox-Foster DL (2007). A metagenomic survey of microbes in honey bee colony collapse disorder. Science.

[30] Rehan SM, Leys R, Schwarz MP (2012). A mid-cretaceous origin of sociality in xylocopine bees with only two origins of true worker castes. PLOS ONE.

[31] Rehan, S. M. Small carpenter bees (Ceratina). Encyclopedia of Social Insects (ed Chris, S.) (Springer, 2020).

[32] Sakagami SF, Maeta Y (1984). Multifemale nests and rudimentary castes in the normally solitary bee *Ceratina japonica* (Hymenoptera: Xylocopinae). J. Kans. Entomol..

[33] Huisken JL, Shell WA, Pare HK, Rehan SM (2021). The influence of social environment on cooperating and conflict in an incipiently social bee, *Ceratina calcarata*. Behav. Ecol..

[34] Rehan SM, Glastad KM, Lawson SP, Hunt BG (2016). The genome and methylome of a subsocial small carpenter bee, Ceratina calcarata. GBE.

[35] Rehan SM (2018). Conserved genes underlie phenotypic plasticity in an incipiently social bee. GBE.

[36] Arsenault SV, Hunt BG, Rehan SM (2018). The effect of maternal care on gene expression and DNA methylation in a subsocial bee. Nat. Commun..

[37] Shell WA (2021). Sociality sculpts similar patterns of molecular evolution in two independently evolved lineages of eusocial bees. Comms. Biol..

[38] Dew RM, McFrederick QS, Rehan SM (2020). Diverse diets with consistent core microbiome in wild bee pollen provisions. Insects.

[39] Lawson SP, Kennedy K, Rehan SM (2021). Pollen composition significantly impacts development and survival of the native small carpenter bee, *Ceratina calcarata*. Ecol. Entomol..

[40] Oppenheimer RL, Shell WA, Rehan SM (2018). Phylogeography and population genetics of the Australian small carpenter bee, *Ceratina australensis*. Biol. J. Linn. Soc..

[41] McFrederick QS, Rehan SM (2018). Wild bee pollen usage and microbial communities co- vary across landscapes. Microb. Ecol..

[42] Rehan SM, Richards MH, Schwarz MP (2010). Sociality in the Australian small carpenter bee *Ceratina (Neoceratina) australensis*. Insectes Sociaux.

[43] Harpur BA, Rehan SM (2021). Connecting social polymorphism to single nucleotide polymorphism: population genomics of the small carpenter bee, *Ceratina australensis*. Biol. J. Linn. Soc..

[44] Neu AT, Allen EE, Roy K (2021). Defining and quantifying the core microbiome: challenges and prospects. PNAS.

[45] Lawson SP, Ciaccio KN, Rehan SM (2016). Maternal manipulation of pollen provisions affects worker production in a small carpenter bee. Behav. Ecol..

[46] Ganeshprasad, D. N., Jani, K., Shouche, Y. S. & Sneharani, A. H. Gut bacterial inhabitants of open nested honey bee, *Apis florea*. Preprint at https://assets.researchsquare.com/files/rs-225332/v1/ddf21abe-2456-4f45-af61-4ba3e81d16e7.pdf?c=1641312753 (2021).

[47] Rothman JA, Cox-Foster DL, Andrikopoulos C, McFrederick QS (2020). Diet breadth affects bacterial identity but not diversity in the pollen provisions of closely related polylectic and oligolectic bees. Insects.

[48] Cohen H, McFrederick QS, Philpott SM (2020). Environment shapes the microbiome of the blue orchard bee, *Osmia lignaria*. Microb. Ecol..

[49] Dew RM, Rehan SM, Schwarz MP (2016). Biogeography and demography of an Australian native bee *Ceratina australensis* (Hymenoptera: Apidae) since the last glacial maximum. J. Hymenopt. Res..

[50] Pinto-Tomás AA (2009). Symbiotic nitrogen fixation in the fungus gardens of leaf-cutter ants. Science.

[51] Walterson AM, Stavrinides J (2015). *Pantoea* insights into a highly versatile and diverse genus within the Enterobacteriaceae. FEMS Microbiol. Rev..

[52] Scheiner R, Strauß S, Thamm M, Farré-Armengol G, Junker RR (2020). The bacterium *Pantoea ananatis* modifies behavioral responses to sugar solutions in honeybees. Insects.

[53] Leonhardt SD, Kaltenpoth M (2014). Microbial communities of three sympatric Australian stingless bee species. Plos ONE.

[54] Bailey, L. & Ball, B. V. Honey Bee Pathology (Academic Press, 1991).

[55] Tham VL (1978). Isolation of *Streptococcus pluton* from the larvae of European honey bees in Australia. Aust. Vet. J..

[56] Bowman J (2006). The genus *Flavobacterium*. Prokaryotes.

[57] Voordouw G (1995). The genus *Desulovibrio*: The centennial. Appl. Environ. Microbiol..

[58] Singaravelen N, Nee’man G, Inbar M, Izhaki I (2005). Feeding responses of free-flying honeybees to secondary compounds mimicking floral nectars. J. Chem. Ecol..

[59] Baracchi D, Marples A, Jenkins AJ, Leitch AR, Chittka L (2017). Nicotine in floral nectar pharmacologically influences bumblebee learning of floral features. Sci. Rep..

[60] Adler LS, Irwin RE (2005). Ecological costs and benefits of defenses in nectar. Ecology.

[61] Bally J (2021). *Nicotiana paulineana*, a new Australian species in *Nicotiana* section Suaveolentes. Aust. Syst. Bot..

[62] Coenye T, Vandamme P (2003). Diversity and significance of *Burkholderia* species occupying diverse ecology niches. Environ. Microbiol..

[63] Levy A, Merritt AJ, Aravena-Roman M, Hodge MM, Inglis TJJ (2008). Expanded range of *Burkholderia* species in Australia. Am. J. Trop. Med. Hyg..

[64] Kaltenpoth M, Flórez LV (2019). Versatile and dynamic symbioses between insects and *Burkholderia* bacteria. Annu. Rev. Entomol..

[65] Foley K, Fazio G, Jensen AB, Hughes WOH (2012). Nutritional limitation and resistance to opportunistic *Aspergillus* parasites in honey bee larvae. J. Invertebr. Pathol..

[66] Yoder JA (2013). Fungicide contamination reduces beneficial fungi in bee bread based on an area-wide field study in honey bee, *Apis mellifera*, colonies. J. Toxicol. Environ. Health Part A.

[67] Yun J-H, Jung M-J, Kim PS, Bae J-W (2018). Social status shapes the bacterial and fungal gut communities of the honey bee. Sci. Rep..

[68] Dew RM, Silva DP, Rehan SM (2019). Range expansion of an already widespread bee under climate change. GECCO.

[69] Cambra M, Capote N, Myrta A (2006). & Llácer, G. Plum pox virus and the estimated costs associated with sharka disease. EPPO Bull..

[70] Roberts JMK, Ireland KB, Tay WT, Paini D (2018). Honey bee-assisted surveillance for early plant virus detection. Ann. Appl. Biol..

[71] Elliott B (2021). Pollen diets and niche overlap of honey bees and native bees in protected areas. BAAE.

[72] Porrini, C. et al. Use of honey bees as bioindicators of environmental pollution in Italy. in Honey bees: estimating the environmental impact of chemicals (eds Devillers, J. & Pham-Delegue, M.-H.) (Taylor & Francis Press, 2002).

[73] Kennedy P, Higginson AD, Radford AN, Sumner S (2018). Altruism in a volatile world. Nature.

[74] Rubin BER, Sanders JG, Turner KM, Pierce NE, Kocher SD (2018). Social behaviour in bees influences the abundance of *Sodalis* (Enterobacteriaceae) symbionts. R. Soc. Open Sci..

[75] Mohr KI, Tebbe CC (2006). Diversity and phylotype consistency of bacteria in the guts of three bee species (Apoidea) at an oilseed rape field. Environ. Microbiol..

[76] Raymann K, Moran NA (2018). The role of the gut microbiome in health and disease of adult honey bee workers. Curr. Opin. Insect Sci..

[77] Amin FAZ (2020). Probiotic properties of *Bacillus* strains isolated from stingless bee (*Heterotrigona itama*) honey collected across Malaysia. Int. J. Envrion. Res. Public Health.

[78] Takeshita K, Kikuchi Y (2017). *Riptortus pedestris* and *Burkholderia* symbiont: an ideal model system for insect-microbe symbiotic associations. Res. Microbiol..

[79] Martinson VG (2011). A simple and distinctive microbiota associated with honey bees and bumble bees. Mol. Ecol..

[80] D’Alvise P (2018). The impact of winter feed type on intestinal microbiota and parasites in honey bees. Apidologie.

[81] Wang L (2019). Dynamic changes of gut microbial communities of bumble bee queens through important life stages. mSystems.

[82] Kapheim KM, Johnson MM, Jolley M (2021). Composition and acquisition of the microbiome in solitary, ground-nesting alkali bees. Sci. Rep..

[83] Abdelazez A (2018). Potential benefits of *Lactobacillus plantarum* as probiotic and its advantages in human health and industrial applications: A review. Adv. Environ. Biol..

[84] Frese SA (2011). The evolution of host specialization in the vertebrate gut symbiont *Lactobacillus reuteri*. PLoS Genet.

[85] Duar RM (2017). Lifestyles in transition: evolution and natural history of the genus *Lactobacillus*. FEMS Microbiol. Rev..

[86] Tejerina MR, Cabana MJ, Benitez-Ahrendts MR (2021). Strains of *Lactobacillus* spp. reduce chalkbrood in *Apis mellifera*. J. Invertebr. Pathol..

[87] Vásquez A (2012). Symbionts as major modulators of insect health: Lactic acid bacteria and honeybees. PLOS ONE.

[88] Voulgari-Kokota A, Steffan-Dewenter I, Keller A (2020). Susceptibility of red mason bee larvae to bacterial threats due to microbiome exchange with imported pollen provisions. Insects.

[89] Steffan SA (2019). Omnivory in bees: Elevated trophic positions among all major bee families. Am. Nat..

[90] Hurst, P. S. Social biology of *Exoneurella tridentata*, an allodapine bee with morphological castes and perennial colonies. Unpublished D. Phil. Thesis (Flinders University, 2001).

[91] Chalita M (2021). Improved metagenomic taxonomic profiling using a curated core gene- based bacterial database reveals unrecognized species in the genus *Streptococcus*. Pathogens.

[92] Rehan SM, Toth AL (2015). Climbing the social ladder: molecular evolution of sociality. Trends Ecol. Evol..

[93] Shell WA, Rehan SM (2018). Behavioral and genetic mechanisms of social evolution: insights from incipiently and facultatively social bees. Apidologie.

[94] Kirby KS (1956). Isolation and characterization of ribosomal ribonucleic acid. Biochem. J..

[95] Bolger AM, Lohse M, Usadel B (2014). Trimmomatic: a flexible trimmer for Illumina sequence data. Bioinformatics.

[96] Li H, Durbin R (2019). Fast and accurate short read alignment with Burrows-Wheeler transform. Bioinformatics.

[97] Li H (2009). The sequence alignment/map format and SAMtools. Bioinformatics.

[98] Tsilimigras MCB, Fodor AA (2016). Compositional data analysis of the microbiome: fundamentals, tools, and challenges. Ann. Epidemiol..

[99] Wood DE, Lu J, Langmead B (2019). Improved metagenomic analysis with Kraken 2. Genome Biol..

[100] Nurk S, Meleshko D, Korobeynikov A, Pevzner PA (2017). metaSPAdes: a new versatile metagenomic assembler. Genome Res.

[101] Altschul SF (1990). Basic local alignment search tool. J. Mol. Biol..

[102] Oksanen, J. et al. Package ‘vegan’. Community Ecology package, version 2, 1–295 (2013).

[103] Hammer Ø, Harper DAT, Ryan PD (2001). PAST: Paleontological statistics software package for education and data analysis. Palaeontol. Electron..

[104] Buchfink B, Xie C, Huson DH (2015). Fast and sensitive protein alignment using DIAMOND. Nat. Methods.

[105] Mina R, Haixu T, Yuzhen Y (2010). FragGeneScan: predicting genes in short and error-prone reads. Nucleic Acids Res..

[106] Kanehisa M, Sato Y, Morishima K (2016). BlastKOALA and GhostKOALA: KEGG tools for functional characterization of genome and metagenome sequences. J. Mol. Biol..

[107] Huerta-Cepas J (2017). Fast genome-wide functional annotation through orthology assignment by eggNOG-mapper. Mol. Biol. Evol..

[108] Love MI, Huber W, Anders S (2014). Moderated estimation of fold change and dispersion for RNA-seq data with DESeq2. Genome Biol..

[109] Langfelder P, Horvath S (2008). WGCNA: an R package for weighted correlation network analysis. BMC Bioinf.

[110] Langfelder, P. & Horvath, S. Tutorials for the WGCNA package. https://horvath.genetics.ucla.edu/html/CoexpressionNetwork/Rpackages/WGCNA/Tutorials/ (2016).

[111] Liaw A, Wiener M (2002). Classification and regression by randomForest. R. N..

[112] Kuhn M (2008). Building predictive models in R using the caret package. J. Stat. Softw..

[113] Paluszynska, A. Structure mining and knowledge extraction from random forest with applications to The Cancer Genome Atlas project. Master’s Thesis (University of Warsaw, 2017).

