# Supplementary material for: Comparative metagenomics reveals expanded insights into intra- and interspecific variation among wild bee microbiomes
Source: Commun Biol. 2022 Jun 17;5:603. doi: 10.1038/s42003-022-03535-1 (PMC9205906; doi:10.1038/s42003-022-03535-1)
Supplement: Supplementary file 1 — Supplementary Information [file 42003_2022_3535_MOESM1_ESM.pdf]

Comparative metagenomics reveals expanded insights into intra- and interspecific variation among wild bee microbiomes

SUPPLEMENTAL FIGURES

(panels being on next page)

SUPPLEMENTARY TABLES AND FIGURES

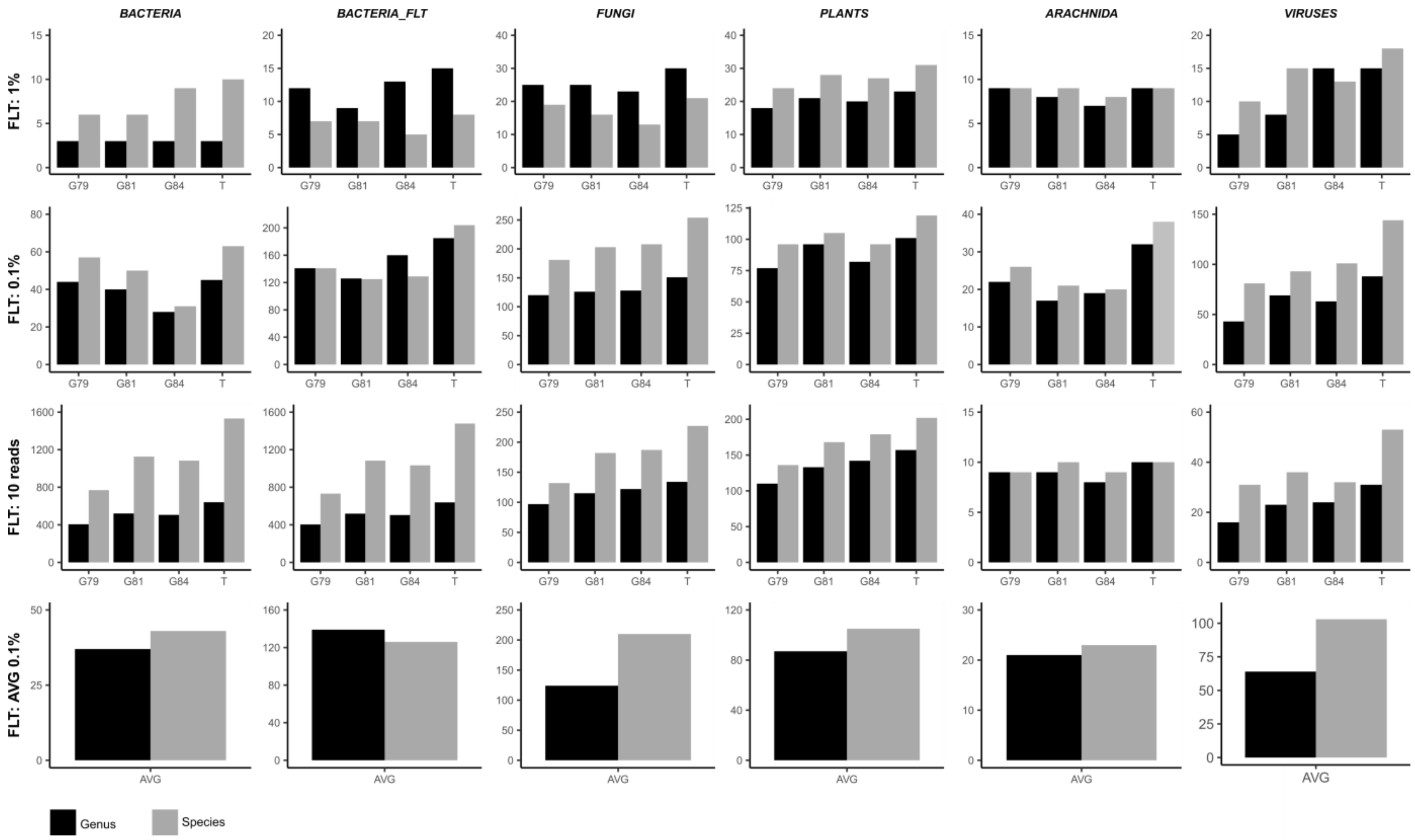

Figure S1. Impact of filtering strategy on the number of Kraken classified genera and species in three *C. calcarata* specimens. Filtering thresholds and 1%, 0.1% and 10 reads are reported for each sample. 'T' represents a total number of unique taxa classified across samples. AVG represents the min 0.1% across all samples of *C. calcarata*. BACTERIA_FLT represents classification report with *Wolbachia* and *Sodalis* excluded.

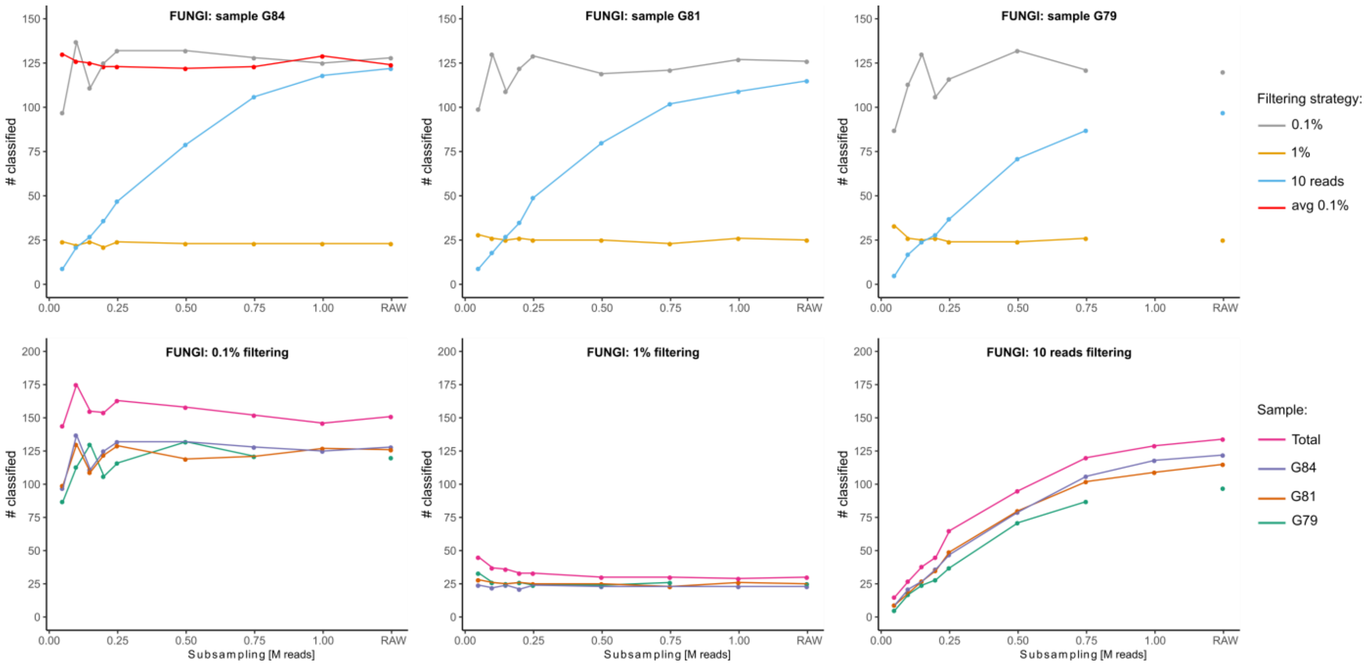

Figure S2. Illustrative example of Kraken subsampling effect on classified genera, in this case Fungi, among *C. calcarata* samples. Upper row of plots depicts per sample strategy, lower panel plots depict per filtering strategy. All tested samples were found to be acceptably within the rarefied range with the 0.1% filtering approach.

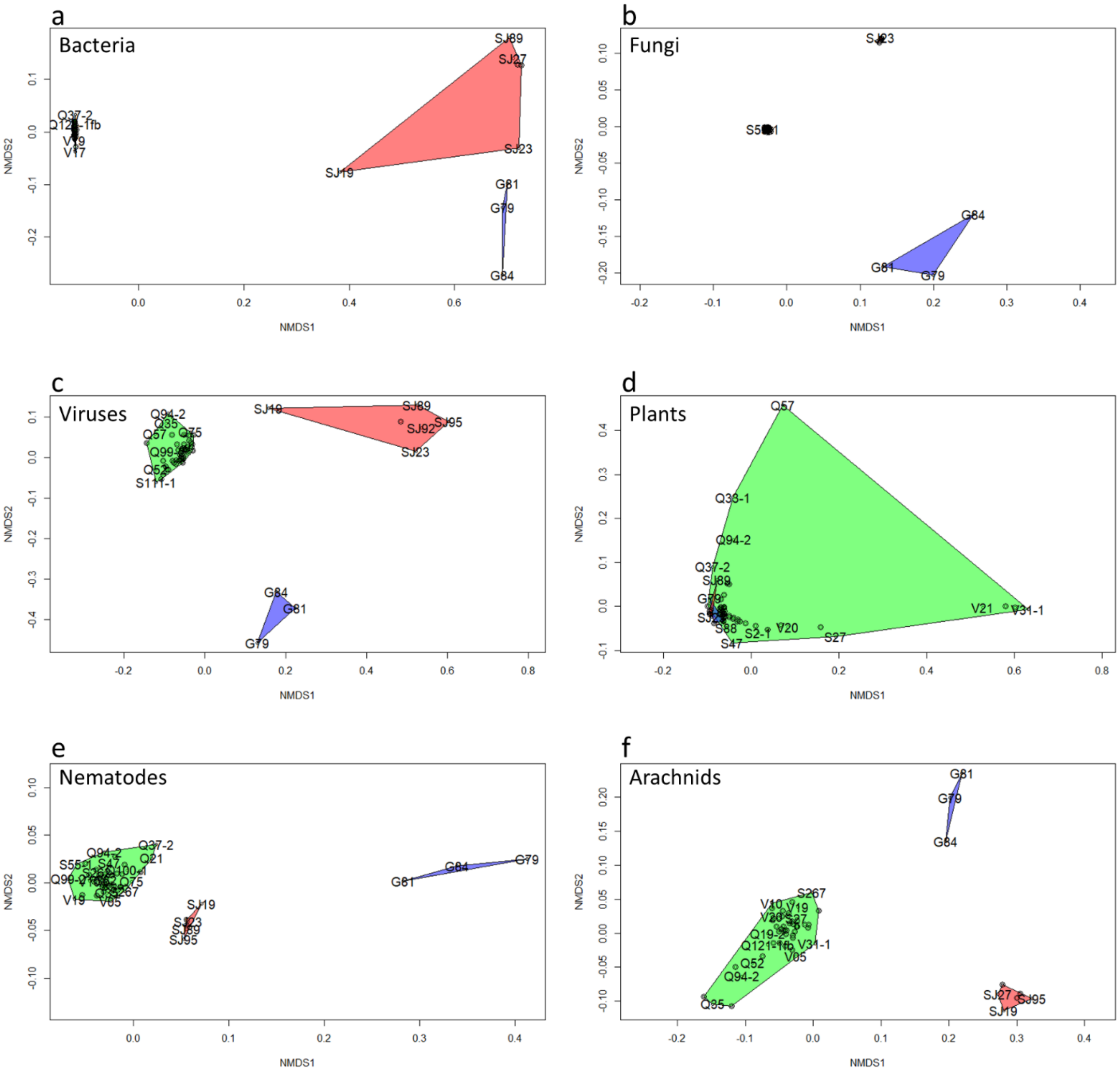

Figure S3 Effects of host species on community composition of six focal groups of families identified in metagenomic data. Nonmetric multidimensional scaling (NMDS) of the community compositions of a) bacteria, b) fungi, c) viruses, d) plants, e) nematodes, and f) arachnids identified in *C. australensis* (green), *C. japonica* (red), and *C. calcarata* (blue). Polygons define community dispersion for each taxonomic group within each host species. In all cases except plants, variation in community composition was significantly affected by host species ($p < 0.001$; **Table S4**)

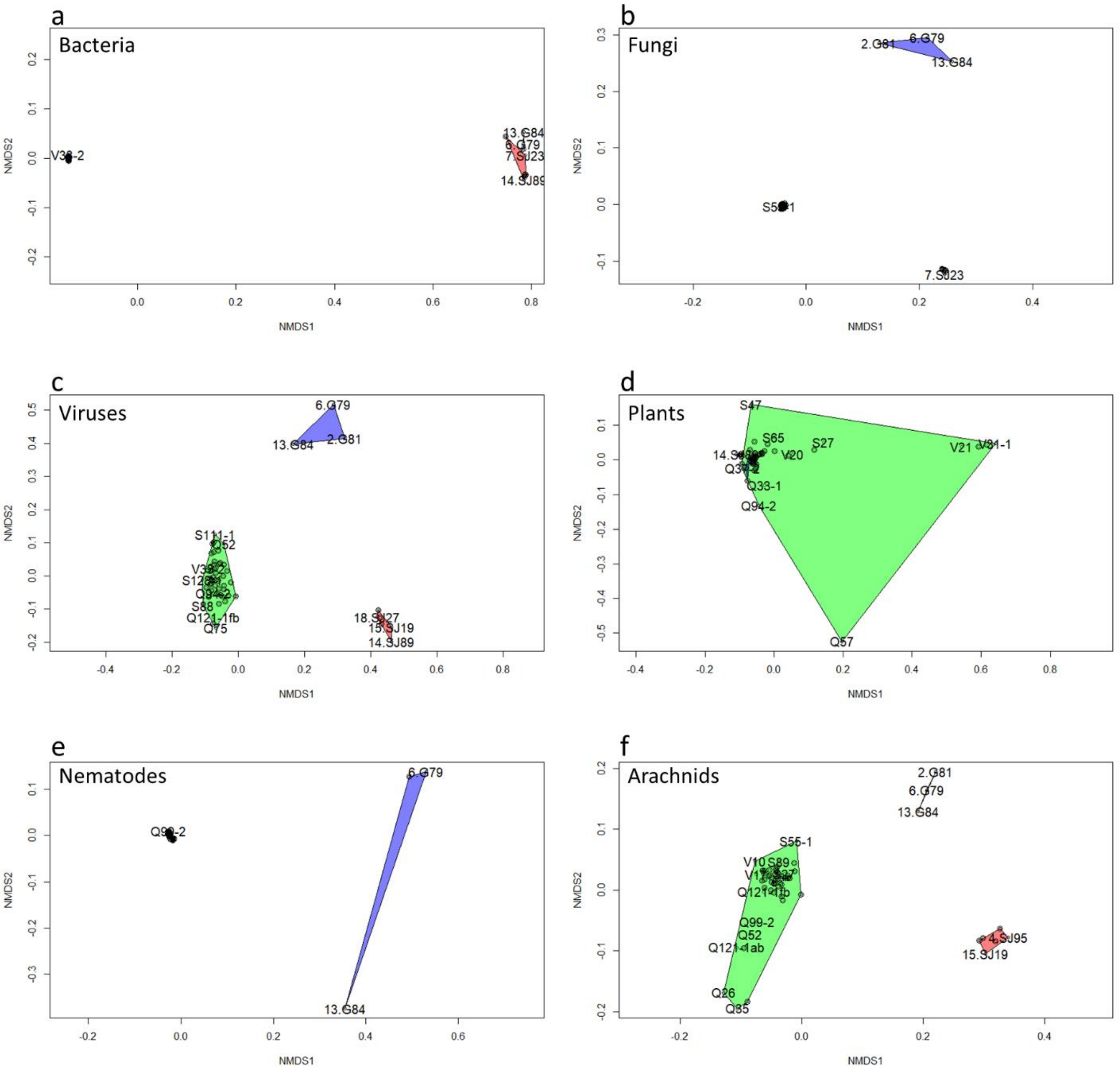

Figure S4 Effects of host species on community composition of six focal groups of genera identified in metagenomic data. Nonmetric multidimensional scaling (NMDS) of the community compositions of a) bacteria, b) fungi, c) viruses, d) plants, e) nematodes, and f) arachnids identified from *C. australensis* (green), *C. japonica* (red), and *C. calcarata* (blue). Polygons define community dispersion for each taxonomic group within each host species. In all cases, variation in community composition was significantly affected by host species ($p \leq 0.047$; **Table S4**).

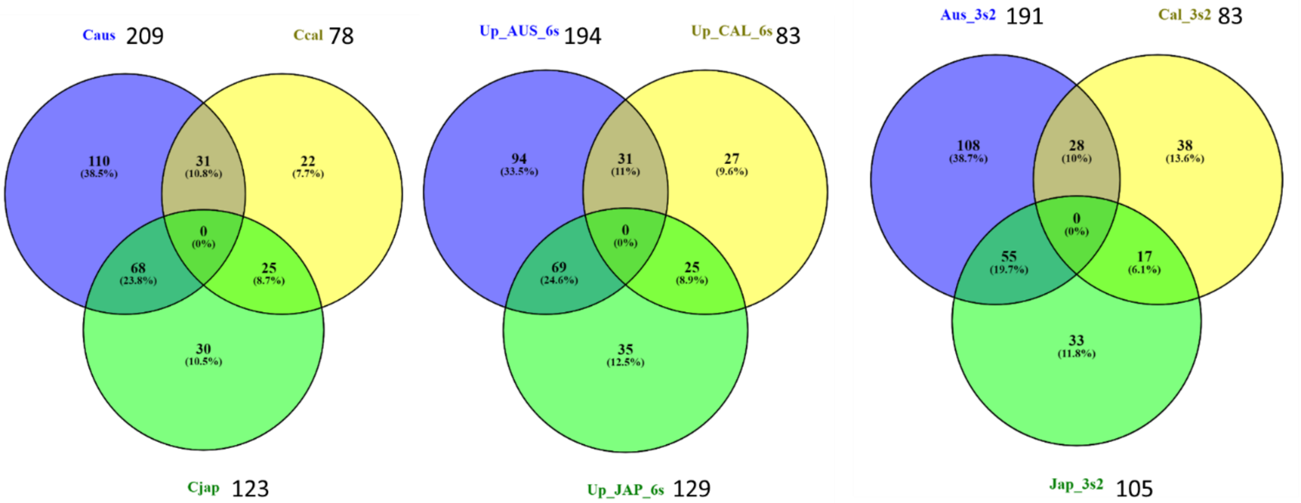

Figure S5. Results of rarefied negative binomial testing of metagenomic count data associated with *C. australensis*, *C. japonica*, and *C. calcarata*. Relative frequencies of differentially occurrent phyla did not vary significantly among tests (Chi square; Chi = 2.35, df = 4, p = 0.67).

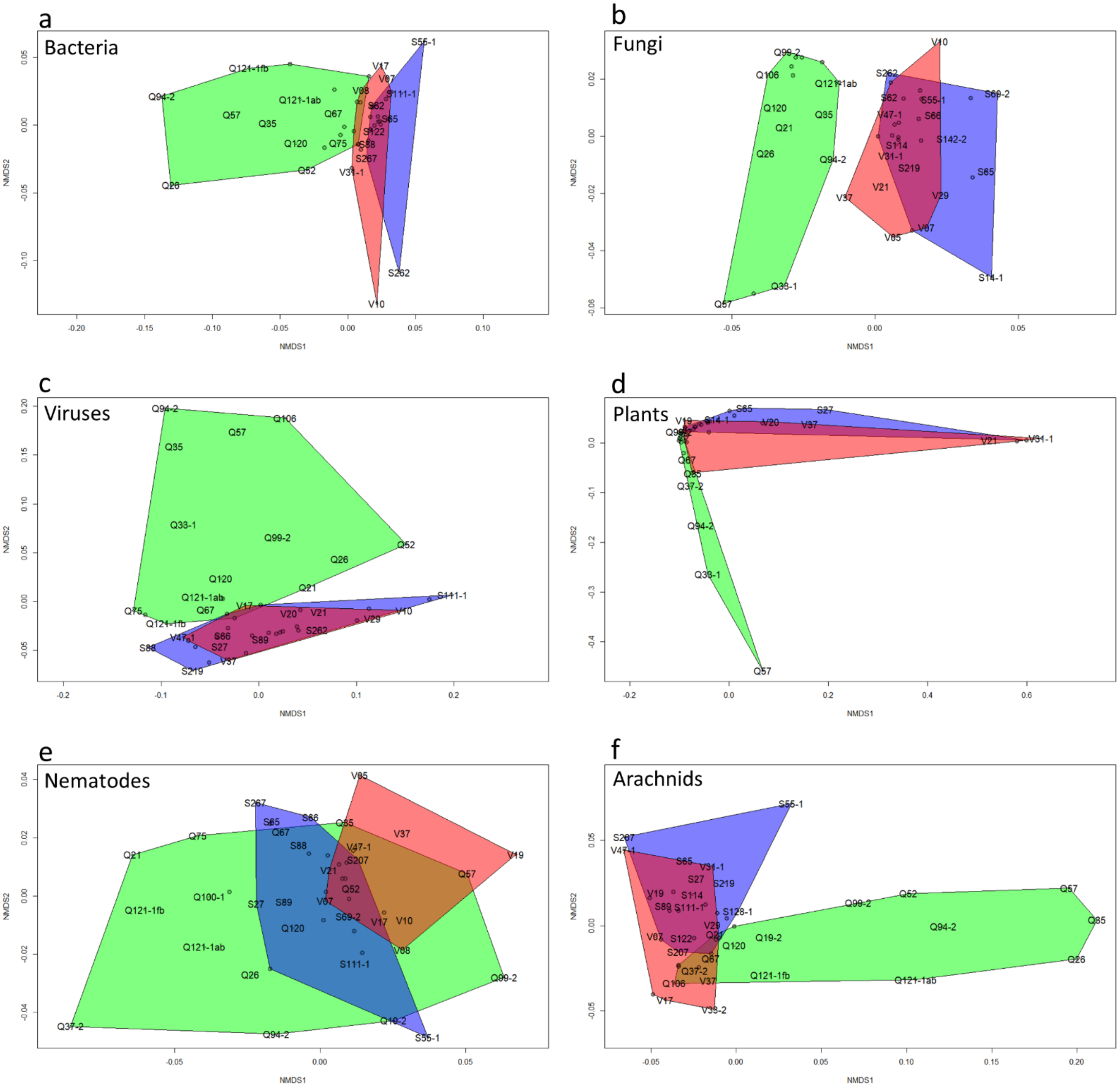

Figure S6. Effects of *C. australensis* population of origin on community composition of six focal groups of families identified in metagenomic data. Nonmetric multidimensional scaling (NMDS) of the community compositions of a) bacteria, b) fungi, c) viruses, d) plants, e) nematodes, and f) arachnids identified from *C. australensis* individuals collected in Queensland (green), Victoria (red), or South Australia (blue). Polygons define community dispersion for each taxonomic group within each population of origin. In all cases, variation in community composition was significantly affected by population of origin ($p \leq 0.005$; **Table S18**).

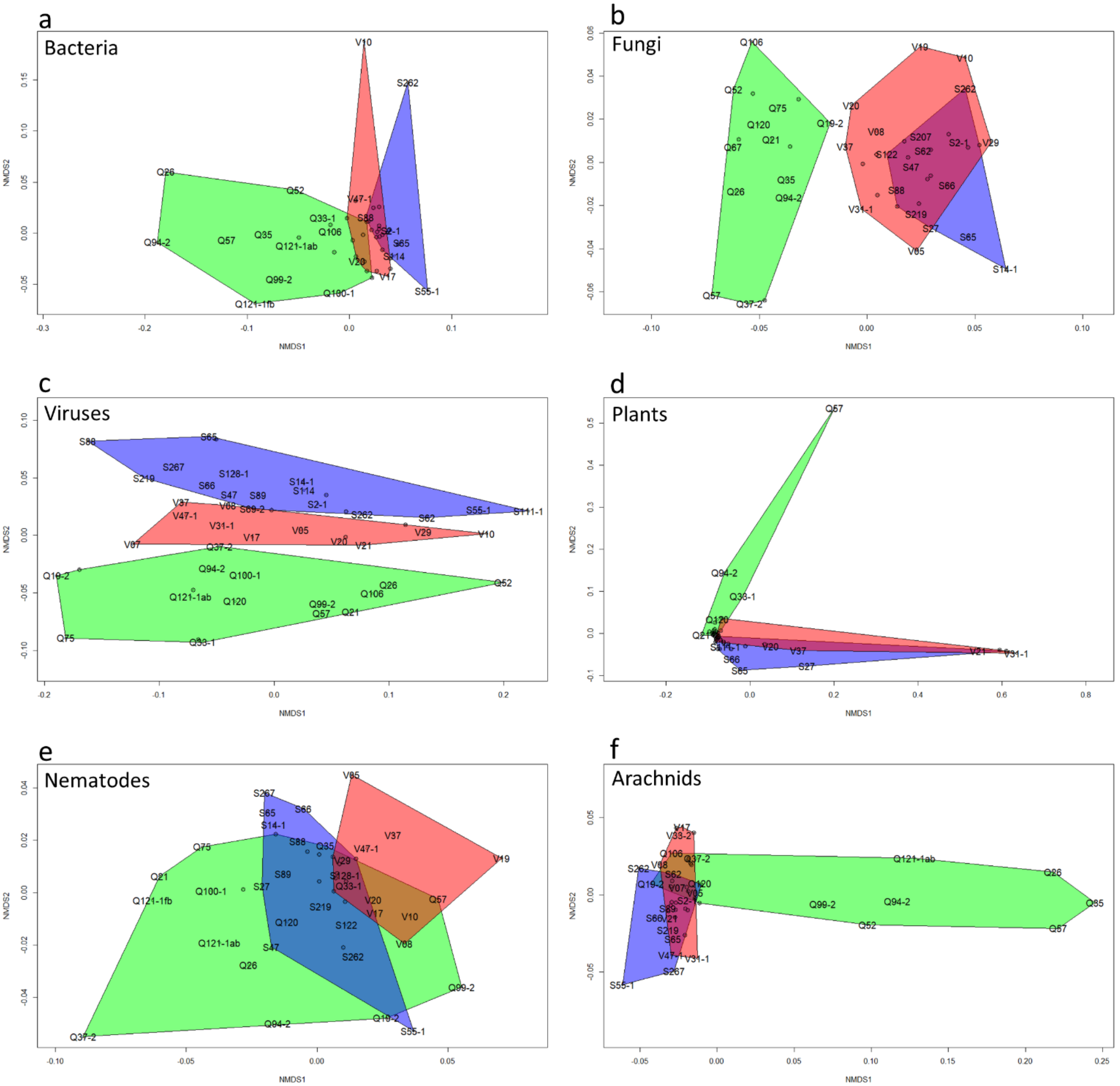

Figure S7. Effects of *C. australensis* population of origin on community composition of six focal groups of genera identified in metagenomic data. Nonmetric multidimensional scaling (NMDS) of the community compositions of a) bacteria, b) fungi, c) viruses, d) plants, e) nematodes, and f) arachnids identified from *C. australensis* individuals collected in Queensland (green), Victoria (red), or South Australia (blue). Polygons define community dispersion for each taxonomic group within each population of origin. In all cases, variation in community composition was significantly affected by population of origin ($p \leq 0.005$; **Table S18**).

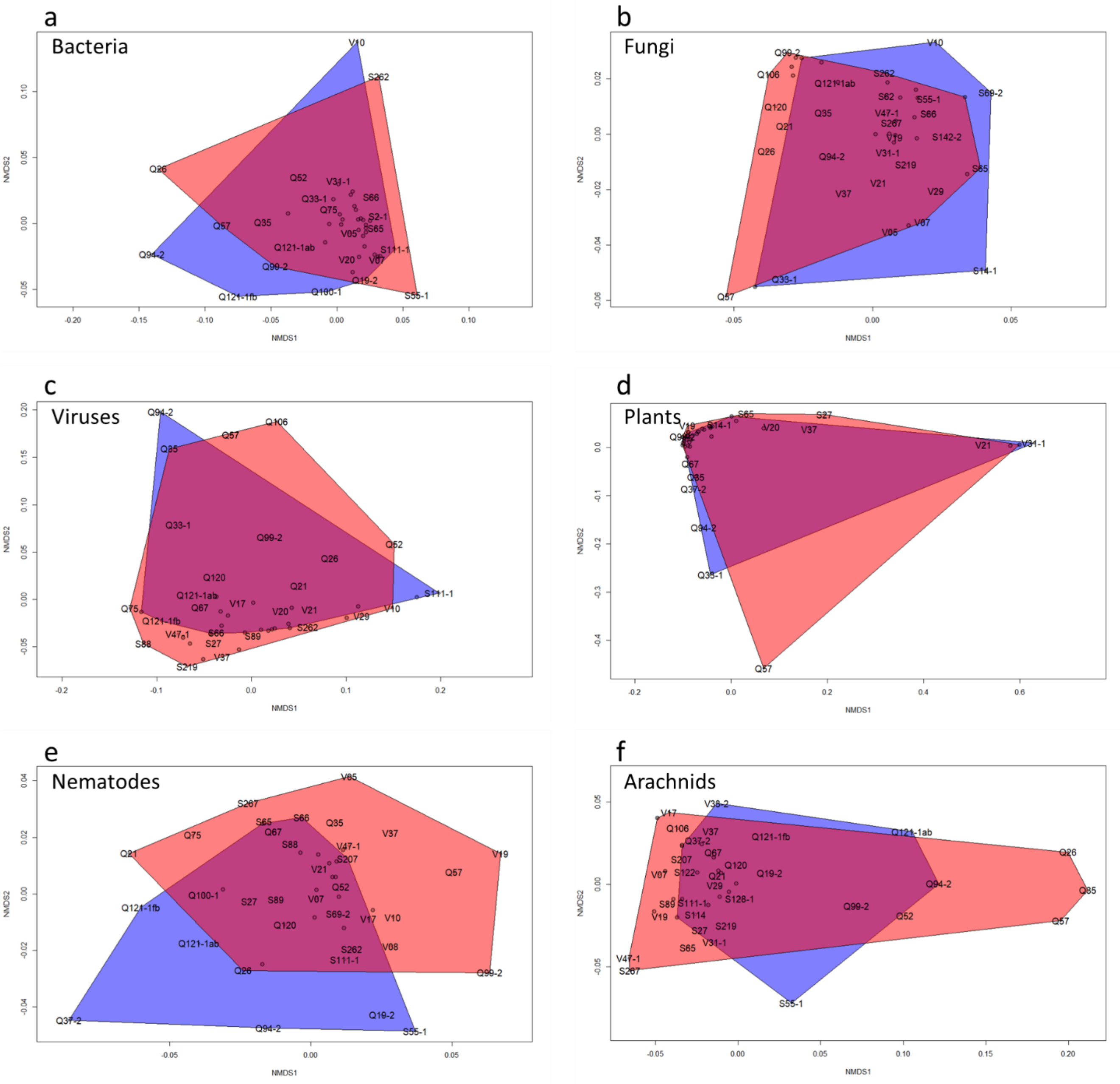

Figure S8. Effects of *C. australensis* sociality on community composition of six focal groups of families identified in metagenomic data. Nonmetric multidimensional scaling (NMDS) of the community compositions of a) bacteria, b) fungi, c) viruses, d) plants, e) nematodes, and f) arachnids identified from *C. australensis* individuals collected from social (blue) or solitary nest types (red). Polygons define community dispersion for each taxonomic group within each population of origin. In all cases, variation in community composition was not significantly affected by population of origin ($p > 0.05$; **Table S23**).

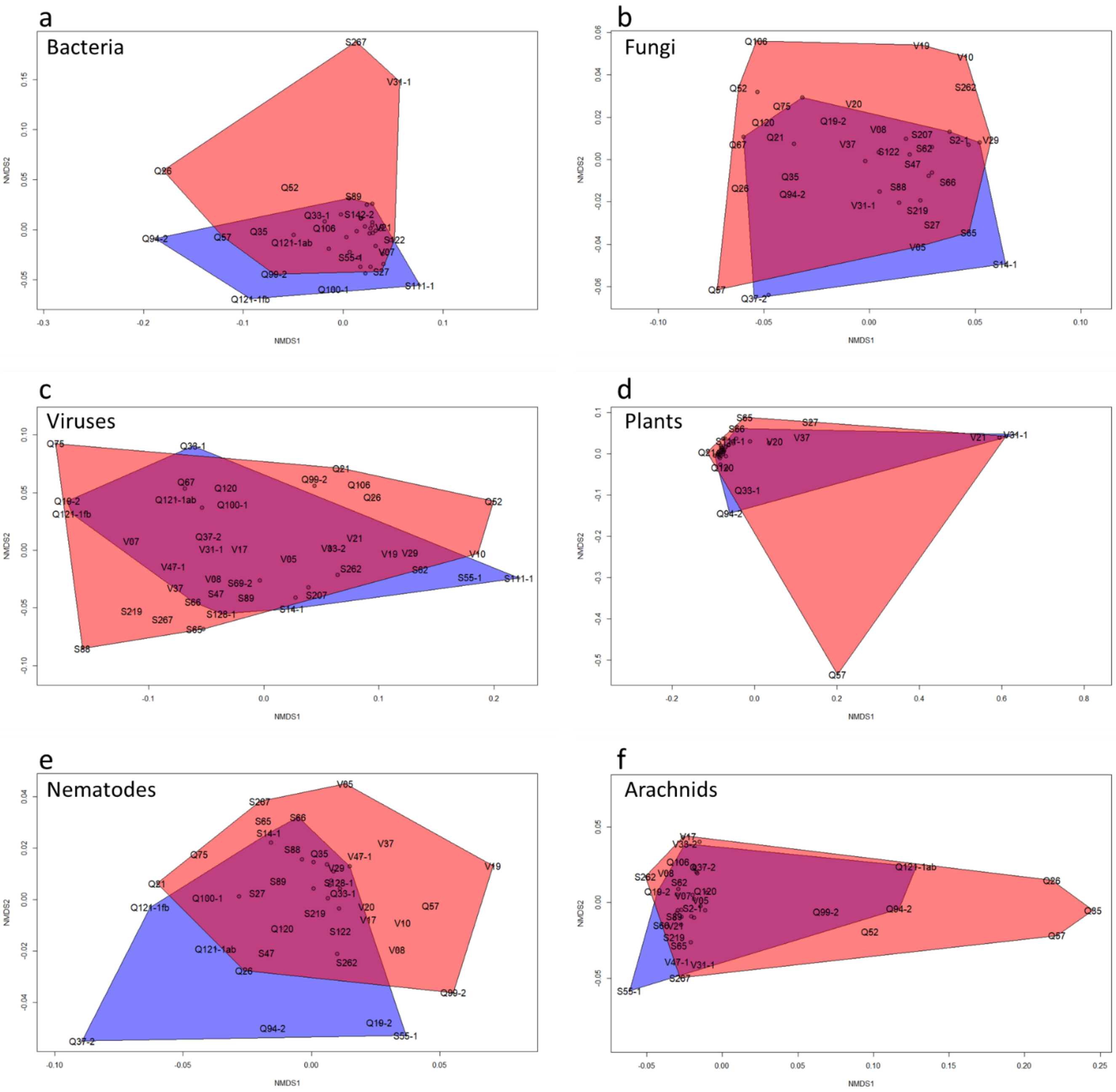

Figure S9. Effects of *C. australensis* sociality on community composition of six focal genera identified in metagenomic data. Nonmetric multidimensional scaling (NMDS) of the community compositions of a) bacteria, b) fungi, c) viruses, d) plants, e) nematodes, and f) arachnids identified from *C. australensis* individuals collected from social (blue) or solitary nest types (red). Polygons define community dispersion for each taxonomic group within each population of origin. In all cases, variation in community composition was not significantly affected by population of origin ($p > 0.05$; **Table S23**).

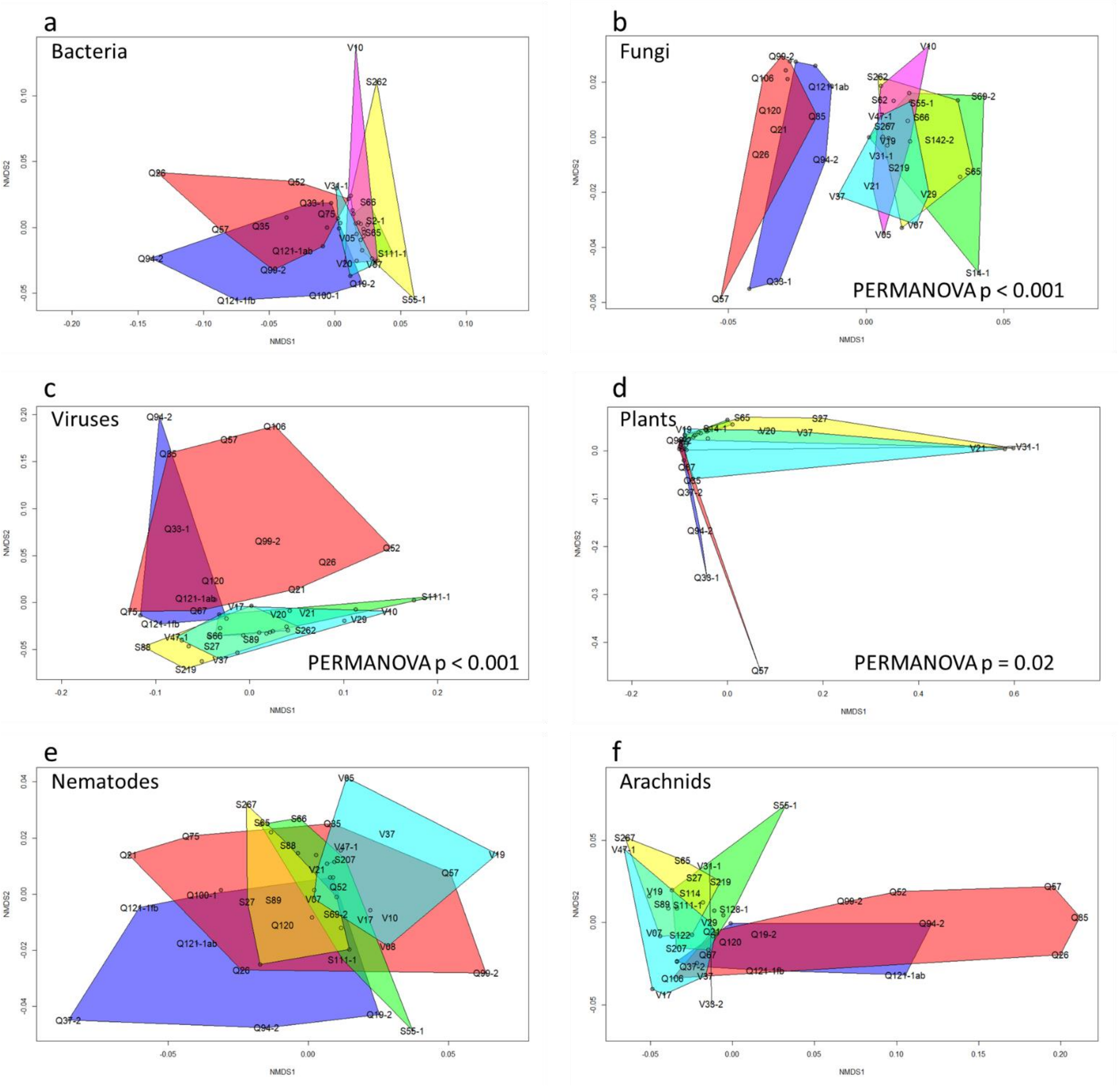

Figure S10. Effects of *C. australensis* sociality and site on community composition of six focal groups of families identified in metagenomic data. Nonmetric multidimensional scaling (NMDS) of the community compositions of a) bacteria, b) fungi, c) viruses, d) plants, e) nematodes, and f) arachnids identified from *C. australensis* individuals collected in Queensland (soc = blue; sol = red), Victoria (soc = magenta; sol = cyan), and South Australia (soc = green; sol = yellow). Polygons define community dispersion for each taxonomic group within each population of origin. Variation in community composition of fungi, viruses, and plants was significantly affected by sociality and site ($p < 0.05$; **Table S26**).

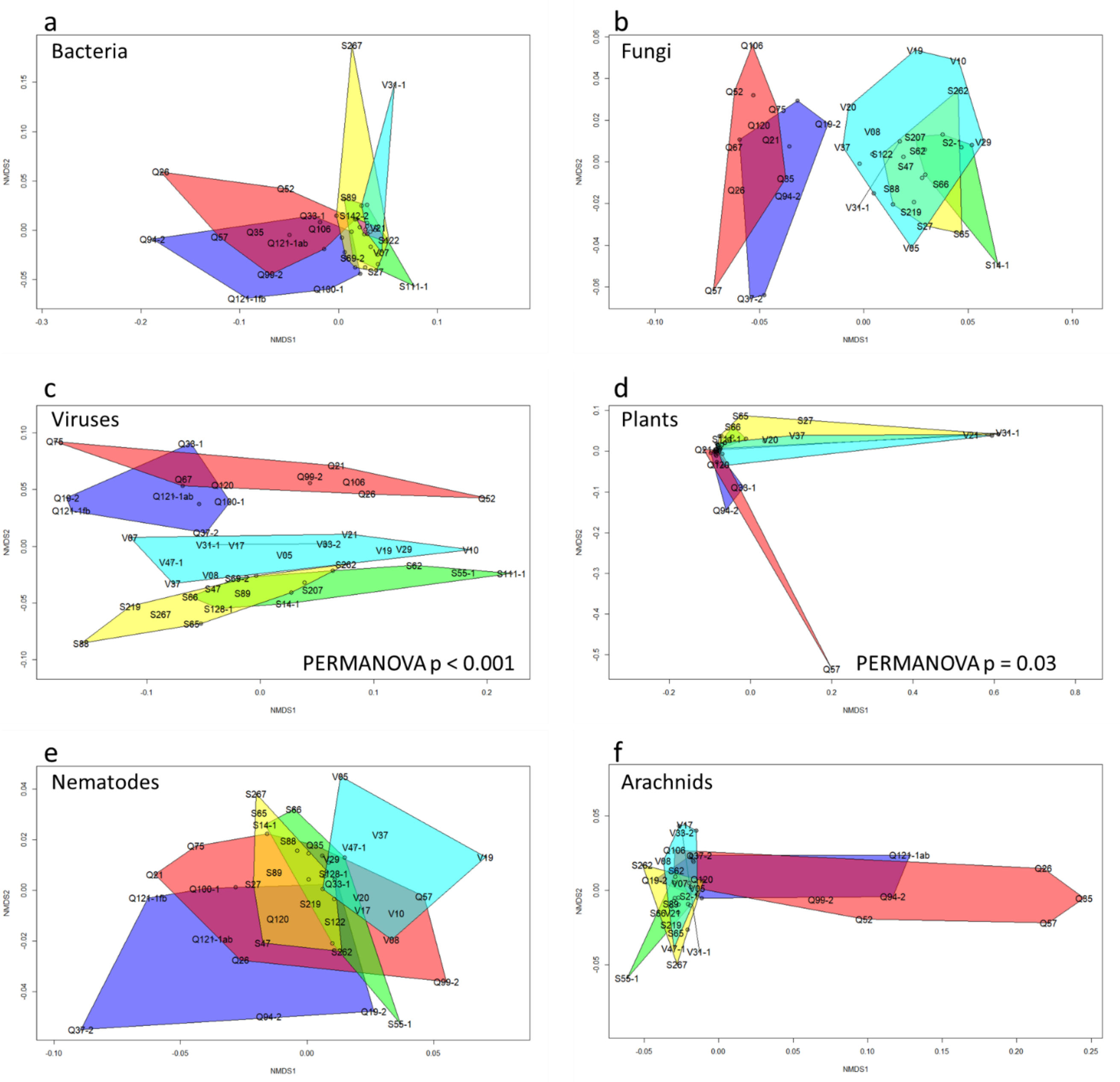

Figure S11. Effects of *C. australensis* sociality and site on community composition of six focal groups of genera identified in metagenomic data. Nonmetric multidimensional scaling (NMDS) of the community compositions of a) bacteria, b) fungi, c) viruses, d) plants, e) nematodes, and f) arachnids identified from *C. australensis* individuals collected in Queensland (soc = blue; sol = red), Victoria (soc = magenta; sol = cyan), and South Australia (soc = green; sol = yellow). Polygons define community dispersion for each taxonomic group within each population of origin. Variation in community composition of viruses and plants was significantly affected by sociality and site ($p < 0.05$; **Table S26**).

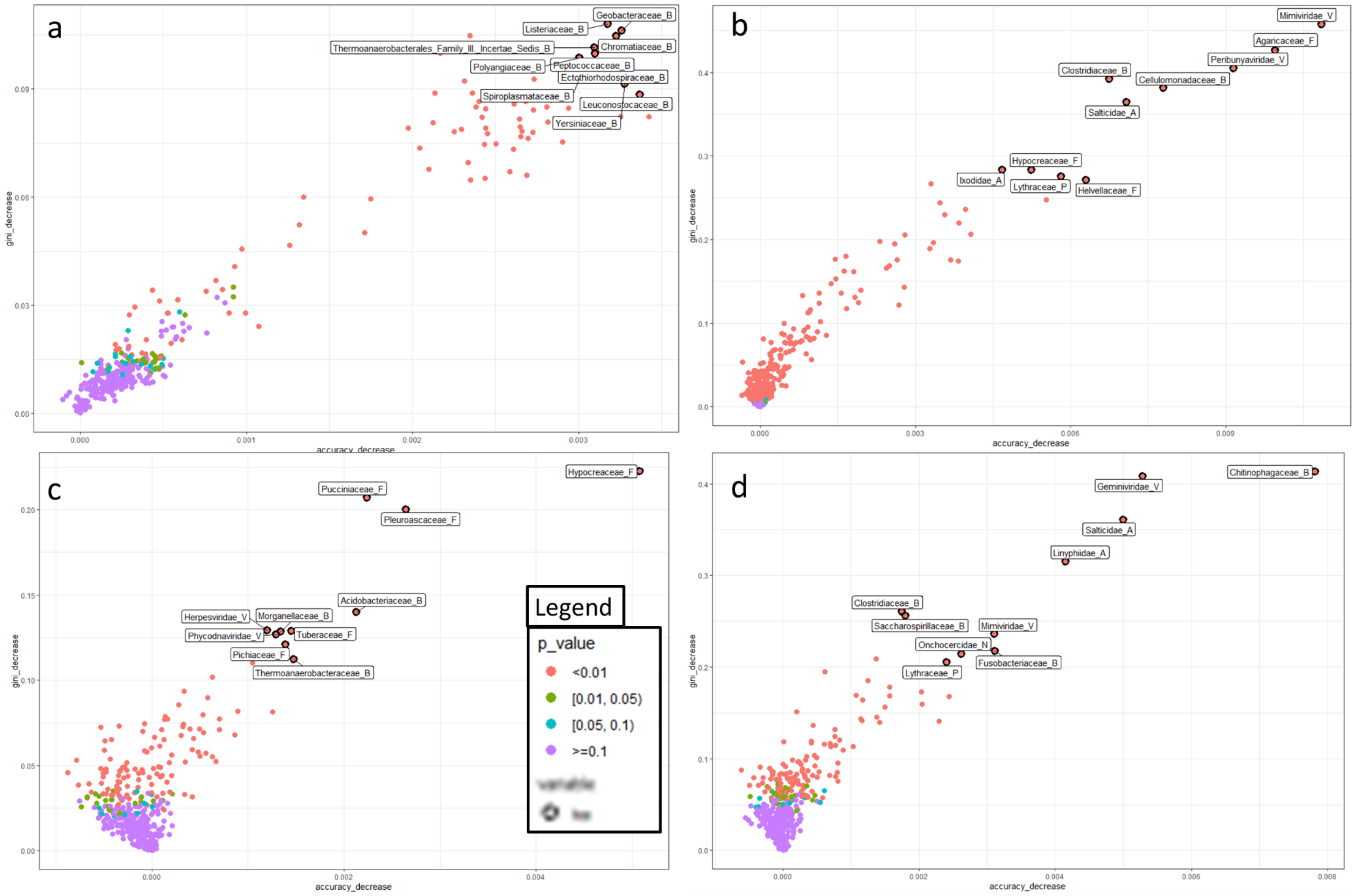
a
Geobacteraceae_B
Listeriaceae_B
Thermoanaerobacterales_Family_III._Incertae_Sedis_B
Chromatiaceae_B
Polyangiaceae_B
Peptococcaceae_B
Ectothiorhodospiraceae_B
Spiroplasmataceae_B
Leuconostocaceae_B
Yersiniaceae_B
gini_decrease
accuracy_decrease
b
Mimiviridae_V
Agaricaceae_F
Peribunyaviridae_V
Clostridiaceae_B
Cellulomonadaceae_B
Salticidae_A
Hypocreaceae_F
Ixodidae_A
Lythraceae_P
Helvellaceae_F
gini_decrease
accuracy_decrease
c
Hypocreaceae_F
Pucciniaceae_F
Pleuroascaceae_F
Acidobacteriaceae_B
Herpesviridae_V
Morganellaceae_B
Phycodnaviridae_V
Tuberaceae_F
Pichiaceae_F
Thermoanaerobacteraceae_B
gini_decrease
accuracy_decrease
Legend
p_value
<0.01
[0.01, 0.05)
[0.05, 0.1)
>=0.1
d
Chitinophagaceae_B
Geminiviridae_V
Salticidae_A
Linyphiidae_A
Clostridiaceae_B
Saccharospirillaceae_B
Mimiviridae_V
Onchocercidae_N
Lythraceae_P
Fusobacteriaceae_B
gini_decrease
accuracy_decrease

Figure S12. Multiway importance plots depicting families of greatest importance for RFC performance accuracy with regard to resolving a) RFC 1 – host species, b) RFC 2 – population of origin, c) RFC 3 – host sociality, and d) RFC 4 – host sociality by population. In each chart, families are plotted by their impact on model accuracy (accuracy_decrease; x) as a product of their overall statistical dispersion (gini_decrease; y). The top 10 most important families are indicated in charts, full datasets are available for each test in **Table S6**.
