# Supplementary material for: Comparative metagenomics reveals expanded insights into intra- and interspecific variation among wild bee microbiomes
Source: Commun Biol. 2022 Jun 17;5:603. doi: 10.1038/s42003-022-03535-1 (PMC9205906; doi:10.1038/s42003-022-03535-1)
Supplement: Supplementary file 2 — Description of Additional Supplementary Files [file 42003_2022_3535_MOESM2_ESM.pdf]

Description of Additional Supplementary Files

File name: Data S1-S30
Description: 30 supplementary data tables containing:

Data S1. Summary of sequencing, mapping, and Kraken classification. Counts of raw and cleaned read pairs, REF_map [%] from bamtools, REF_unmap, REF_unmap [%], and reads classified by Kraken vs nt database (#class_Krk) are provided for all samples.

Data S2. Summary results of BLAST analyses of metaspades outputs. Significantly orthologous hits to metagenomic contigs assembled from *C. australensis* (A-FM), *C. calcarata* (FO-GB), and *C. japonica* (GD-HA) are presented.

Data S3. Core bacterial, fungal, viral, and plant genera identified among three *Ceratina* host species. For each species, core taxa are those which are present in >50% of samples and with >1% relative abundance. Bacterial taxa were calculated without *Wolbachia* and *Sodalis*.

Data S4. Beta dispersion ANOVA and Adonis PERMANOVA testing of effects of host species on metagenomic community diversity at both the family and genus levels for each group.

Data S5. Total accounting of random forest classifier performance accuracy across species (A-K), population (M-V), and sociality testing (Z-CA). Training sample set size, test number, accuracy with 95% confidence interval bounds, no information rate (random sort rate), McNemar's Test p-value, and by-bin performance accuracy are provided where applicable. Below overall test performance results are the top 20 most important taxa for RFC modelling in rows 46-67. In the case of species identity (RFC1), focal module associations from WGCNA for those most important taxa are provided (full results of WGCNA analyses located in Data S11-S13)

Data S6. Outputs of RandomForestExplainer analyses summarizing importance of each taxon with regard to model accuracy. Families are sorted by 'gini_decrease,' a measure of overall model impact, followed by p-value (indicating families were used for node splitting more often than by chance). Results of species (A-H), population of origin (J-Q), sociality across populations (S-Z), and sociality within populations (AB-AI) are provided.

Data S7. Results of simper testing to assess overall contributions of families or genera to pairwise bray-curtis dissimilarity between species. The top ten most explanatory phyla are reported at both the family and genus levels for all data and across all major taxonomic bins (e.g. Bacteria, Fungi, Viruses, etc).

Data S8. Total results of GLM testing among species: *Ceratina australensis*, *C. calcarata*, and *C. japonica*. Group, family, log 2-fold change (Log2FC), and p-value adjusted for multiple testing (padj) associated with each condition drawn from pairwise results extracts are provided.

Data S9. Total results of GLM testing among species: *Ceratina australensis*, *C. calcarata*, and *C. japonica*. Group, genus, log 2-fold change (Log2FC), and p-value adjusted for multiple testing (padj) associated with each condition drawn from pairwise results extracts are provided.

Data S10. Significant results of DESeq2 re-testing of rarefied family read data to check for sample size effects. Rarefaction did not have a significant effect on detection of significantly differentially abundant taxa among species.

Data S11. Summary output of WGCNA for host species *Ceratina australensis*. All analyzed taxa, their annotation, module color, correlation with and significance for *C. australensis*, are indicated in columns A-E. Taxon membership value in each module and associated p-values for all additional modules are then specified in columns F through AX. Module columns are ordered by degree of significance of association with *C. australensis* (regardless of positive or negative correlation), with most significant to the left (F), least significant to the right (AX). Rows are sorted alphabetically by module.

Data S12. Summary output of WGCNA for host species *Ceratina japonica*. All analyzed taxa, their annotation, module color, correlation with and significance for *C. japonica*, are indicated in columns A-E. Taxon membership value in each module and associated p-values for all additional modules are then specified in columns F through AX. Module columns are ordered by degree of significance of association with *C. japonica* (regardless of positive or negative correlation), with most significant to the left (F), least significant to the right (AX). Rows are sorted alphabetically by module.

Data S13. Summary output of WGCNA for host species *Ceratina calcarata*. All analyzed taxa, their annotation, module color, correlation with and significance for *C. calcarata*, are indicated in columns A-E. Taxon membership value in each module and associated p-values for all additional modules are then specified in columns F through AX. Module columns are ordered by degree of significance of association with *C. calcarata* (regardless of positive or negative correlation), with most significant to the left (F), least significant to the right (AX). Rows are sorted alphabetically by module.

Data S14. Output details for the top species-associated modules for *C. australensis* (A-F), *C. japonica* (H-XX), and *C. calcarata* (XY-XZ). Family ID, family-to-species significance (GS) and p-value (GS_p), taxon module membership (MM) and p-value (MM_p), and indication of module HUB Status (>0.9 GS and >0.9 MM) are provided.

Data S15. Results of functional enrichment (KEGG) analysis determined using bacterial taxa detected among *C. australensis, C. japonica* and *C. calcarata*. Enriched terms have been sorted into columns indicating unique or shared status by host species (A-G).

Data S16. Core bacterial, fungal, viral, and plant genera identified among three *Ceratina australensis* populations of origin. For each population, core taxa are those which are present in >50% of samples and with >1% relative abundance. Bacterial taxa were calculated without *Wolbachia* and *Sodalis*.

Data S17. Plant pathogenic families and genera detected in Australian populations of honeybees during Roberts et al. 2018 and in *C. australensis* metagenomic data in the current study.

Data S18. Beta dispersion ANOVA and Adonis PERMANOVA testing of effects of population of origin on metagenomic community diversity at both the family and genus levels for each group.

Data S19. Results of simper testing to assess overall contributions of families or genera to pairwise bray-curtis dissimilarity between *C. australensis* populations. The top ten most explanatory phyla are reported at both the family and genus levels for all data and across all major taxonomic bins (e.g. Bacteria, Fungi, Viruses etc) for Queensland vs South Australia (A-H), Queensland vs Victoria (K-R) and South Australia vs Victoria (U-AB).

Data S20. Total results of GLM testing among *C. australensis* populations: Queensland, Victoria, and South Australia. Group, Family, Log 2-fold change (Log2FC), and p-value adjusted for multiple testing (padj) associated with each condition drawn from pairwise results extracts are provided.

Data S21. Total results of GLM testing among *C. australensis* populations: Queensland, Victoria, and South Australia. Group, Genus, Log 2-fold change (Log2FC), and p-value adjusted for multiple testing (padj) associated with each condition drawn from pairwise results extracts are provided.

Data S22. Results of functional enrichment (KEGG) analysis determined using bacterial and fungal phyla, as well as just the bacterial genus *Burkholderia*, detected among *C. australensis* populations of origin. Enriched terms have been sorted into columns indicating unique or shared status by host population for bacteria (A-G), fungi (L-R), and *Burkholderia* (W-AC).

Data S23. Beta dispersion ANOVA and Adonis PERMANOVA testing of effects of population of origin on metagenomic community diversity at both the family and genus levels for each group.

Data S24. Total results of GLM testing of metagenomic family data binning by sociality among *C. australensis* populations. No phyla were significant at $p < 0.05$.

Data S25. Total results of GLM testing of metagenomic genus data binning by sociality among *C. australensis* populations. No phyla were significant at $p < 0.05$.

Data S26. Beta dispersion ANOVA and Adonis PERMANOVA testing of effects of population of origin on metagenomic community diversity at both the family and genus levels for each group.

Data S27. Total results of GLM testing of sociality by population. Group, family, log 2-fold change (Log2FC), and p-value adjusted for multiple testing (padj) associated with each condition drawn from pairwise results extracts are provided.

Data S28. Total results of GLM testing of sociality by population. Group, genus, log 2-fold change (Log2FC), and p-value adjusted for multiple testing (padj) associated with each condition drawn from pairwise results extracts are provided.

Data S29. Catalog of bee taxa and respective studies (including current) included in comparative analyses of bacterial abundance. Three species of *Ceratina* from current study were compared to 35 additional bees for a total dataset of 38 taxa. Full compared data can be found in Data S30.

Data S30. Results of comparative analysis assessing bacterial genera diversity and relative abundances as detected in *C. australensis* populations (B-J), among host *Ceratina* species (K-V) and across 35 additional bee taxa in previous studies (W-CT). Where available, core microbiome status, relative abundance and prevalence data are provided for each bee species.
