# Supplementary material for: Comparative metagenomics reveals expanded insights into intra- and interspecific variation among wild bee microbiomes
Source: Commun Biol. 2022 Jun 17;5:603. doi: 10.1038/s42003-022-03535-1 (PMC9205906; doi:10.1038/s42003-022-03535-1)
Supplement: Supplementary file 4 — Reporting Summary [file 42003_2022_3535_MOESM4_ESM.pdf]

nature portfolio

Corresponding author(s): Sandra M. Rehan

Last updated by author(s): Nov 2, 2021

Reporting Summary

Nature Portfolio wishes to improve the reproducibility of the work that we publish. This form provides structure for consistency and transparency in reporting. For further information on Nature Portfolio policies, see our Editorial Policies and the Editorial Policy Checklist.

Statistics

For all statistical analyses, confirm that the following items are present in the figure legend, table legend, main text, or Methods section.

n/a	Confirmed	
☐	☒	The exact sample size (*n*) for each experimental group/condition, given as a discrete number and unit of measurement
☐	☒	A statement on whether measurements were taken from distinct samples or whether the same sample was measured repeatedly
☐	☒	The statistical test(s) used AND whether they are one- or two-sided<br>*Only common tests should be described solely by name; describe more complex techniques in the Methods section.*
☐	☒	A description of all covariates tested
☒	☐	A description of any assumptions or corrections, such as tests of normality and adjustment for multiple comparisons
☐	☒	A full description of the statistical parameters including central tendency (e.g. means) or other basic estimates (e.g. regression coefficient) AND variation (e.g. standard deviation) or associated estimates of uncertainty (e.g. confidence intervals)
☐	☒	For null hypothesis testing, the test statistic (e.g. *F*, *t*, *r*) with confidence intervals, effect sizes, degrees of freedom and *P* value noted<br>*Give P values as exact values whenever suitable.*
☒	☐	For Bayesian analysis, information on the choice of priors and Markov chain Monte Carlo settings
☐	☒	For hierarchical and complex designs, identification of the appropriate level for tests and full reporting of outcomes
☒	☐	Estimates of effect sizes (e.g. Cohen's *d*, Pearson's *r*), indicating how they were calculated

Our web collection on statistics for biologists contains articles on many of the points above.

Software and code

Policy information about availability of computer code

Data collection	Data used in this study were secured in part through the use of the publicly available UniProt, NCBI databases and any associated code or web based user interfaces.
Data analysis	We used and cited only publicly available code to analyze data for this study, including FastQC, Trimmomatic, bwa mem, samtools, BLASTn, Kraken2, metaSPAdes, PAST v 4.06, DIAMOND, FragGeneScan, GhostKOALA, and eggNOG-mapper, along with R script packages DESeq2, WGCNA, randomForest, RandomForestExplainer, and Vegan.

For manuscripts utilizing custom algorithms or software that are central to the research but not yet described in published literature, software must be made available to editors and reviewers. We strongly encourage code deposition in a community repository (e.g. GitHub). See the Nature Portfolio guidelines for submitting code & software for further information.

Data

Policy information about availability of data

All manuscripts must include a data availability statement. This statement should provide the following information, where applicable:

- Accession codes, unique identifiers, or web links for publicly available datasets
- A description of any restrictions on data availability
- For clinical datasets or third party data, please ensure that the statement adheres to our policy

All newly generated metagenomic data used in this study can be freely accessed via NCBI BioProject number PRJNA407923

Field-specific reporting

Please select the one below that is the best fit for your research. If you are not sure, read the appropriate sections before making your selection.

☐ Life sciences ☐ Behavioural & social sciences ☒ Ecological, evolutionary & environmental sciences

For a reference copy of the document with all sections, see nature.com/documents/nr-reporting-summary-flat.pdf

Ecological, evolutionary & environmental sciences study design

All studies must disclose on these points even when the disclosure is negative.

Study description	Comparative analysis of three wild bee metagenomes (Apidae: Xylocopinae: Ceratinini) to examine variations in microbiomes by host species, population, and sociality and to compare the core Ceratina microbiome to those of 35 additional bee species.
Research sample	We secured metagenomic data from three small carpenter bee species for which genomes have recently been sequenced and are publicly available on the NCBI database. These species are Ceratina australensis, collected from three populations in Australia; Ceratina japonica, collected from Sapporo, Japan; and Ceratina calcarata, collected in Durham, New Hampshire in the USA.
Sampling strategy	These species of Ceratina have been particularly well studied, and are each emerging models for the study of social evolution and behavioral ecology in wild bees. Each species is represented by at least N = 3 individuals (C. calcarata) through N = 51 individuals (C. australensis) to allow for calculation of average taxonomic abundance at both the species and - in C. australensis - population levels.
Data collection	gDNA samples were extracted by WAS and sent for sequencing at Genome Quebec. Resulting raw data were then processed at Genome Quebec and further metagenomic analyses were then performed by WAS and SMR.
Timing and spatial scale	Although samples were collected over the course of three years, timing between sample collections is not expected to have a meaningful impact on results of analysis. Samples from each species were collected during relatively small windows, and species are drawn from highly isolated geographic points of origin (Northeast North America, Japan, and Southeast Australia). The effects of these varied points of origin are considered thoroughly in the manuscript.
Data exclusions	No data were excluded from our analyses
Reproducibility	When necessary, analyses of genomic data were re-run to confirm consistency of results
Randomization	Taxonomic abundance data were first binned and analyzed by host species (i.e. C. japonica vs C. australensis, vs C. calcarata); then C. japonica and C. calcarata were set aside, and C. australensis data were re-assessed by population of origin (Victoria vs Queensland vs South Australia), sociality (solitary vs social), and population of origin x sociality. We then drew on published datasets to compare bacterial taxa identified in 35 additional bee species to those identified in our Ceratina species.
Blinding	Blinding was not possible nor necessary for our study. Our findings are based on the results of comprehensive comparative analyses among all relevant samples. Research awareness of sample types was thus critical for proper analysis.

Did the study involve field work? ☒ Yes ☐ No

Field work, collection and transport

Field conditions	Atmospheric conditions varied greatly from one sampling location to the next but did not have a meaningful impact on our sampling or study design.
Location	Sample collections were performed around Durham, New Hampshire; Sapporo, Japan; and South Australia, Australia.
Access & import/export	*Describe the efforts you have made to access habitats and to collect and import/export your samples in a responsible manner and in compliance with local, national and international laws, noting any permits that were obtained (give the name of the issuing authority, the date of issue, and any identifying information).*
Disturbance	Samples were collected primarily through sweep netting, thus disturbance caused by this study was minimal

Reporting for specific materials, systems and methods

We require information from authors about some types of materials, experimental systems and methods used in many studies. Here, indicate whether each material, system or method listed is relevant to your study. If you are not sure if a list item applies to your research, read the appropriate section before selecting a response.

Materials & experimental systems

n/a	Involved in the study	
☒	☐	Antibodies
☒	☐	Eukaryotic cell lines
☒	☐	Palaeontology and archaeology
☐	☒	Animals and other organisms
☒	☐	Human research participants
☒	☐	Clinical data
☒	☐	Dual use research of concern

Methods

n/a	Involved in the study	
☒	☐	ChIP-seq
☒	☐	Flow cytometry
☒	☐	MRI-based neuroimaging

Animals and other organisms

Policy information about studies involving animals; ARRIVE guidelines recommended for reporting animal research

Laboratory animals	This study did not involve animals kept or reared in a laboratory
Wild animals	This study did not involve the observation or live capture of wild animals
Field-collected samples	Bees of the species Ceratina calcarata (North America), Ceratina japonica (Japan), and Ceratina australensis (Australia) were captured either on the wing via target sweep netting or from their nesting substrate (typically a hollow stick) in field sites located in their countries of origin. After positive visual identification, individuals were quickly killed via flash freezing in liquid nitrogen for temperature controlled transportation back to our lab in Durham, New Hampshire, USA. In the lab, females were identified during dissections on dry ice for use in genomic sample extraction.
Ethics oversight	As the bees used in this study are invertebrate insects of no extinction concern, no ethical approval or guidance was required for the collection or manipulation of these samples.

Note that full information on the approval of the study protocol must also be provided in the manuscript.
